# Reconstructing the Network of Horizontal Gene Exchange in Bacteria to Differentiate Direct and Indirect Transfers

**DOI:** 10.1093/gbe/evag099

**Published:** 2026-04-18

**Authors:** Michael Sheinman, Tommaso Stentella, Paul Etheimer, Florian Massip, Peter F Arndt

**Affiliations:** Department of Physics of Complex Systems, Weizmann Institute of Science, Rehovot, Israel; Institute for Advanced Studies, Sevastopol State University, Sevastopol, Russia; Max Planck Institute for Molecular Genetics, Berlin, Germany; Centre for Computational Biology (CBIO), Mines Paris, PSL University, Paris 75006, France; Computational Oncology, Institut Curie, PSL University, Paris 75005, France; INSERM, U1331, Paris 75005, France; Centre for Computational Biology (CBIO), Mines Paris, PSL University, Paris 75006, France; Computational Oncology, Institut Curie, PSL University, Paris 75005, France; INSERM, U1331, Paris 75005, France; Max Planck Institute for Molecular Genetics, Berlin, Germany

**Keywords:** horizontal gene transfer, evolution, bacteria, network

## Abstract

Horizontal gene transfer (HGT) plays a central role in bacterial evolution. Yet, its large-scale dynamics and underlying network structure remain poorly characterized. We present a theoretical framework that models HGT as a continuous stochastic process over a network of bacterial genera and analyze its genomic footprint via the distribution of exact sequence matches shared across taxa—the match length distribution (MLD). We show that different evolutionary regimes imprint distinct statistical signatures on the MLD: single episodic gene transfer events yield exponential distributions, while continuous sustained HGT processes lead to power-law tails. The power-law exponent is analytically linked to the topology of the transfer network, distinguishing between intra-clade transfers and hub-mediated dissemination. Empirical MLDs derived from bacterial genomes recapitulate these predicted patterns. Moreover, we find that defining a genus-specific “transferability” parameter that governs pairwise HGT rates, and incorporating a high-transferability hub, accurately reproduces the observed data. Our approach provides a general framework for inferring hidden structure in genomic horizontal transfer processes, enabling quantitative analysis of microbial evolution.

Significance Horizontal gene transfer (HGT) shapes bacterial evolution, yet its large-scale dynamics remain difficult to infer from genomic data. We show that the distribution of exact sequence matches shared between bacterial genomes contains quantitative signatures of different HGT regimes and of the underlying transfer network topology. This framework enables inference of hidden structure in microbial gene exchange directly from genome sequence data.

## Introduction

The most influential concepts in biological evolution—such as the phylogenetic tree (Darwin 1859) and the molecular clock (Zuckerkandl and Pauling 1965)—are not readily applicable to the bacterial domain of life. In bacteria, evolutionary relationships are often better described by a *phylogenetic web* rather than by a strictly bifurcating tree (Doolittle 1999; Bapteste et al. 2009), and molecular divergence fails to consistently correlate with speciation time (Ochman et al. 1999). These deviations from canonical models are largely attributed to the pervasive influence of *horizontal gene transfer* (HGT) (Ochman et al. 2000).

The widespread occurrence of HGT has reshaped our understanding of microbial evolution, giving rise to the concept of a web of life (Doolittle 1999; Soucy et al. 2015). While the existence of HGT is well established, its large-scale organization and the statistical signatures it leaves in genome sequences remain poorly understood. It was found that some bacterial lineages function as phylogenetic hubs, receiving and donating genes more extensively than others (Treangen and Rocha 2011 ). However, the relative importance of such transfer hubs—and, more generally, of indirect transfer paths compared to direct exchanges—has never been characterized (Corel et al. 2016). Previous studies have used different measures of genomic similarities between different bacteria to identify and quantify homologous recombination and gene transfer (Novichkov et al. 2004; Dixit et al. 2015; Ravenhall et al. 2015; Sakoparnig et al. 2021). Such measures of shared homologous sequences can then be used to build weighted HGT networks (see *e.g.* Kunin et al. 2005; Dagan et al. 2008; Khaledian et al. 2020; Li et al. 2020; Redondo-Salvo et al. 2020; Shapiro et al. 2023; López Sánchez and Lafond 2024). In these and other studies, usually, when the shared homology cannot be explained by conservation, an edge between the nodes is established. Using such an approach, direct and indirect transfers cannot be distinguished. Furthermore, edges represent a similarity measure but not the direct HGT rate.

**Fig. 1.**
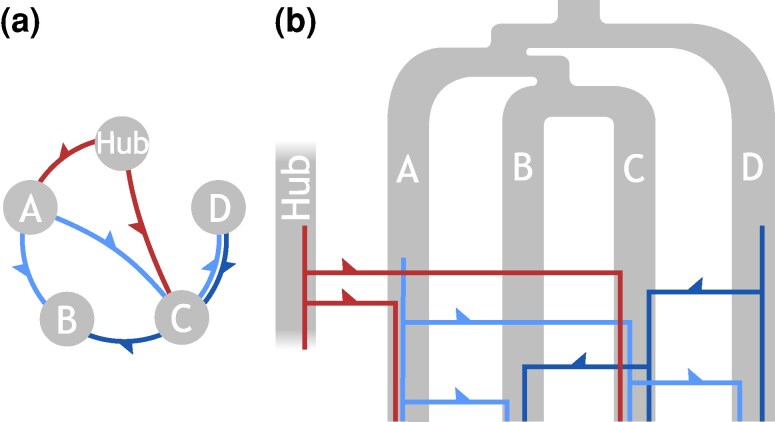
Illustration of a) HGT dissemination scenarios between four distant taxa, A,B,C and *D*, and an un-sampled “hub” taxon, resulting in b) three phylogenetic trees (red and blue lines). Thick grey lines represent the speciation history of the taxa. We assume that all the speciation events occurred long before the recent HGT events which dominate the long exact matches between the genomes of the taxa. The HGTs are shown as arrows in a). The total (vertical, to the most recent common ancestor) length of each HGT resulting tree in b) is defined as *τ*.

In the present study we analyze maximal exact sequence matches shared by genomes to infer the HGT network. Long exact matches have been observed between highly divergent bacterial taxa, such as *Escherichia* and *Salmonella* (Battistuzzi et al. 2004; Šorfová et al. 2008; Kumar et al. 2017). Since their presence cannot be explained by vertical inheritance under typical bacterial mutation rates (Drake 1991; Didelot et al. 2016; Duchêne et al. 2016; Zhao et al. 2019), they are the remnant of past HGT. Such long matches are easy to find and they provide a strong signal of recent gene transfers and other processes (Smillie et al. 2011; Harris and Nielsen 2013; Massip and Arndt 2013; Massip et al. 2015; Massip et al. 2016; Evans et al. 2020; Groussin et al. 2021; Sheinman et al. 2021; Dmitrijeva et al. 2024; Sheinman et al. 2024). However, the presence of a long exact match between two distant genomes does not necessarily imply a direct transfer between the two i→j . It could have been mediated by a third taxon (i→k→j) or acquired independently from a shared donor (i←k→j), as shown in Fig. 1. These possibilities naturally suggest viewing HGT as a *network-level process*, where gene flow occurs both directly and indirectly through interconnected taxa. To disentangle direct and indirect transfers, we develop a quantitative model of HGT as a *continuous stochastic process on a genus-level network*. In our model, edges represent the direct HGT rate, in contrast to a measure of similarity. Note that bacteria of a genus might live in different environments (Cordero and Polz 2014); nevertheless, our model considers only the network of bacterial taxa (genera) and does not differentiate between bacteria within a genus.

To explore this idea, we analyze the *match length distributions* (MLDs) of maximal exact sequence matches shared among sets of n≥2 genera. Specifically, we consider $m_{s}(r)$, the number of maximal exact sequence matches of length *r* per comparison of n=|s| genomes drawn from *n* different genera. This quantity is the main object of analysis in the present study. Extending previous work (Sheinman et al. 2021, 2024), we show that the shape of the MLD encodes key information about the structure of the underlying HGT process. The MLD reflects the interplay between HGT events, which introduce new exact sequence matches between donor and recipient genomes, and mutations, which progressively break long matches into shorter ones. Roughly speaking, the prefactor of this distribution reflects the rate of HGT relative to the mutation rate, while the shape of the distribution $m_{s}(r)$ contains information about the structure of the HGT network within the genus set *s*. A single transfer event produces an exponential distribution; multiple discrete events generate mixtures of exponentials; and a continuous HGT process yields a *power-law tail* of the form $m(r)∼r^{-\alpha }$. Crucially, the exponent *α* reflects the topology of the transfer network.

Notably, the network can be represented in a minimal way by assigning to each genus a characteristic *transferability* parameter, such that the HGT rate between any two genera is given by the product of their transferabilities. This simple representation leads to concrete, testable predictions for the statistical properties of exact sequence matches shared between genomes.

In the following sections, we first quantify the extent of multi-genera exact matches (Section 2). We then derive analytical predictions for match-length distributions under a continuous HGT model and show how indirect transfers via a hub modify the scaling exponents (Section 3). Then we fit the model to all observed MLDs (Section 4). Finally, we discuss mechanistic interpretation and broader implications (Section 5).

## Identifying HGT Events Affecting Multiple Genera

To investigate the exchange of genetic material, we began by identifying exact sequence matches—DNA segments that are identical across genomes from multiple genera within the *Enterobacteriaceae* family and the *Serratia* genus. Following (Sheinman et al. 2021), we restricted our analysis to very long matches (≥1,000 bp): such a high threshold was chosen to ensure we exclude matches resulting from evolutionary conservation (Sheinman et al. 2024). We first detected exact matches shared between pairs of genomes and then extended this analysis to identify regions shared among larger sets of genera (*i.e.* sequences identical in *n* genera, with *n* ranging from 3 to 8).

Under neutral expectations (if matches shared between 2 genera A and B were independent from the matches shared between 2 other genera C and D), the number of shared matches should decline rapidly as the number of compared genera increases. Surprisingly, we observed a substantial number of long exact matches shared among three or more species. For example, on average, about 5% of the *Escherichia* genome participates in long exact matches shared with *any* one of the eight genera considered, while 0.39% of the *Escherichia* genome is shared by all eight genera considered (i.e. present in at least one genome in each of the eight genera), whereas under the independence-of-matches hypothesis this number should be much lower (of the order of $5{\%}^{7}≃10^{-9}\ll 0.39\%$). Other genera have similar fractions (see Supplemental Fig. S1). As shown in Supplemental Fig. S2, genes located within these long shared regions are enriched in mobile genetic elements (Partridge et al. 2018). This confirms that those matches are the result of HGT events and not due to contamination or artifacts of the method. It is important to note that obtained function annotations represent mostly recent HGT events and/or highly preserved genes.

**Fig. 2.**
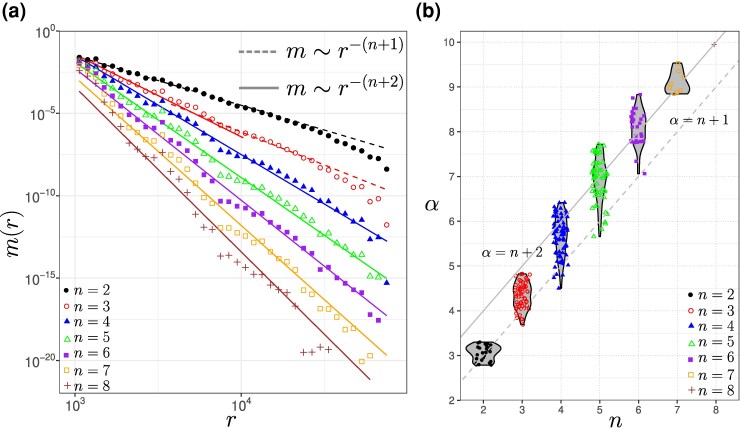
a) Means of MLDs of all comparisons of pairs (n=2), trios (n=3), quartets (n=4) *etc*. The dashed line represents the power-law scaling $r^{-(n+1)}$, while the solid line represents the power-law scaling $r^{-(n+2)}$. Green lines is fitting with sum of two (for n=10) or three (for n=9) exponents. The insets represent the distribution of the power-laws *α* for all comparisons with a given *n* using the method of moments. b) Violin plot of the power-law estimates of the MLDs for all comparisons of pairs (n=2), trios (n=3), quartets (n=4) *etc*. Dashed line represents α=n+1 (only transfers within the set) and solid line represents α=n+2 (transfers via a hub outside of the set).

To gain further insight into the HGT processes, we calculated the distribution of the lengths of matches shared by different sets of genera (see Methods for details). In (Sheinman et al. 2021) we showed that the analysis of the MLD obtained by comparing pair of bacterial species allowed to measure their exchange rate. Thus we tested whether we could apply the same methodology to matches shared by a larger set of species. To our surprise, the MLDs constructed from matches shared by more than two species followed power-law distributions with exponent different from the α=−3 values obtained in our first study (see Fig. 2a). Indeed, the exponent of the power law decreases with the number of species considered (see Fig. 2b).

In the following, we propose a simple model that explains these empirical observations and demonstrate that we can use this model to disentangle the contributions of direct and indirect transfers and measure direct and indirect transfer exchange rates between the eight genera considered in our study.

## The Model and its Analysis

In this section we present the model and derive analytical results for the expected MLD for a given HGT network. Before doing so, it is instructive to analyze a single HGT event spreading in a set of taxa *s* and the shape of its MLD.

### A Single HGT Event

Consider a mobile DNA sequence of a certain length *L* that spreads through the set *s* of taxa. Any dissemination scenario (see Fig. 1a) of such mobile element can be presented as a phylogenetic tree with n=|s| leaves (see Fig. 1b). Defining the total length of this tree by τ≪1 and assuming a constant mutation rate, defined as 1 in our time units, the probability that a bp along the DNA segment is identical for all set members is $e^{-\tau }$. Then, the expected number of maximal exact matches of length r≪L decays exponentially with *r* (Ziff and McGrady 1985; Arndt 2019):


(1) $$m(r\;|\;\tau )=L\tau^{2}e^{-\tau r}.$$

Therefore, for a single HGT that has disseminated in all the studied genera, we expect the length distribution of the exact matches shared by all genomes to be exponential. As a consequence, when several HGT events occur, they should generate an MLD distributed as a mixture of several exponential distributions whose parameter depends on *τ*.

Assuming that HGT is a frequent process, we expect that *τ* has a certain continuous density $P_{s}(\tau )$ for a given HGT network. Then, the MLD of this set is given by


(2) $$m_{s}(r)=L\int_{0}^{\infty }\tau^{2}e^{-r\tau }P_{s}(\tau )d\tau =L{\tilde{P}}_{s}^{\prime \prime }(r)$$

where ${\tilde{P}}_{s}(r)=\int_{0}^{\infty }e^{-r\tau }P_{s}(\tau )d\tau$ is the Laplace transform of $P_{s}(\tau )$. One can see that the length distribution of exact sequence matches is related to the Laplace transform of the distribution of the total length of the phylogenetic trees of the HGT events. Hence, studying the MLD allows us to evaluate the density $P_{s}(\tau )$. In the following, we show that $P_{s}(\tau )$ informs us on the structure of the HGT network. We then study empirical observations of $m_{s}(r)$, to validate the model and fit its parameters for considered genera.

### Continuous HGT Model

The central insight is that exact-match lengths *r* reflect the distribution of total divergence times *τ*, which in turn depends on the HGT network. By modeling HGT as a continuous-time process over a weighted network, we can derive ${\tilde{P}}_{s}(\tau )$ and thus the MLD $m_{s}(r)$ analytically. To do so we model HGT via a weighted network where nodes are bacterial taxa (we use genera in this article, but in this section we keep it more general). A pair of taxa that exchange DNA via HGT are connected by an edge whose weight represents the effective rate (per bp) of HGT between the taxa pair. Effective rates take into account that the transferred gene is not present in 100% of the genomes of the donor taxon and is not transferred to 100% of genomes of the recipient taxon. We assume that all transfers are Poisson processes with different rates. Our analysis focuses on very long exact matches ≥1,000 bp shared by distant taxa. Under realistic bacterial mutation rates, matches of this length typically indicate very recent horizontal transfer, as mutations would rapidly disrupt such long segments of perfect identity over evolutionary time. Below this threshold, however, exact matches may frequently arise from strongly conserved domains rather than recent transfer, which would violate the assumptions of our analysis. Restricting the analysis to matches longer than 1,000 bp therefore minimizes such spurious signals and ensures that the detected matches reflect recent HGT events. Throughout the paper we define time units so that the mutation rate μ=1, and all inferred effective HGT rates are given in units of a typical bacterial mutation rate.

We consider a network of taxa exchanging genetic material that belongs to a mobilome (a collection of all mobile elements) (Siefert 2009; Carr et al. 2021) of a given length. The exchange rate per bp from taxon *i* to taxon *j* is denoted by $\rho_{ij}$ and is assumed to be the same for all elements composing the mobilome. It is possible to compute the analytical solution for a general asymmetric transfer rate matrix (see Equations (S5, S13) in the Supplemental Results S.1), but the solution is then difficult to fit to empirical data. We assume that every genus *i* is characterized by its transferability $\gamma_{i}$, such that the transfer matrix is symmetric and rank-1, given by $\rho_{ij}=\gamma_{i}\gamma_{j}$. Interestingly, this simpler, special case is sufficient to fit the empirical data. We find that it is useful to obtain asymptotic results in the large *r* limit. For the considered system the asymptotic formula is very close to the exact one but has the advantage of being easier to interpret.

#### Asymptotic Results

For any connected set *s*, in the asymptotic regime of large *r* the MLD of the set depends only on the transfer rates within the set (see Supplemental Results, Section S.1.4). The explicit closed form solution of the MLD $m_{s}^{d}(r)$ (*d* for transfers only within the set *s*) can be obtained as shown in Section S.1.4: in a *s*-set of HGT with |s|=n taxa with transfer rates $\rho_{ij}$ the MLDs are given by


(3) $$\begin{array}{rl} m_{s}^{d}(r) & =\frac{L_{d}}{(n-2)!r^{n+1}}\sum_{i_{1}j_{1}\in s}\rho_{i_{1}j_{1}}\sum_{i_{2}j_{2}\in s\setminus j_{1}}\rho_{i_{2}j_{2}}\cdots \\ & \cdots \sum_{i_{n-1}j_{n-1}\in s\setminus j_{1}\setminus j_{2}\cdots \setminus j_{n-2}}\rho_{i_{n-1}j_{n-1}}. \end{array}$$

One can see that in the asymptotic regime the MLD of the set *s* depends only on the HGT rates within the set *s*. $L_{d}$ is the size of the mobilome that is exchanged directly between the considered genera. The scaling law for the long-match asymptotics is


(4) $$m_{s}^{d}(r)∼\frac{1}{r^{\alpha }}$$

where α=n+1=|s|+1. For pairwise comparisons, n=2, one gets the known result α=n+1=3 (Sheinman et al. 2021). As we show below, the prediction α=n+1 for n>2 does not match the empirical data. This discrepancy suggests that for larger genera sets *s*, HGT events are increasingly dominated by an additional, indirect HGT pathway that involves organisms outside the focal set. In the following, we show that such pathway can be explained by a common unobserved network hub.

#### Adding a Strong Hub to the Network

**Fig. 3.**
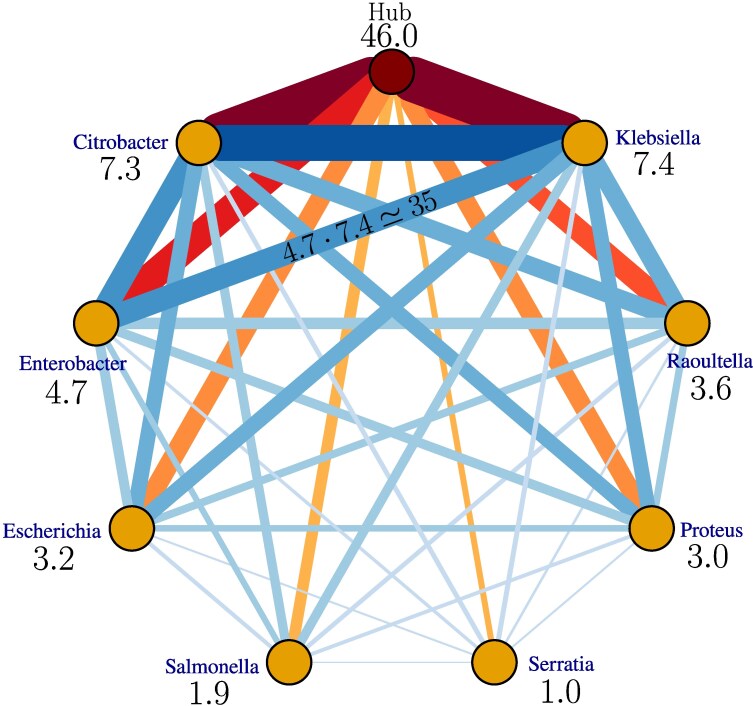
Inferred network of 8 bacteria genera. Thickness of the lines and color intensity is proportional to HGT rate $\rho_{ij}$. The edges from the hub are presented with the shades of red and scaled down by a factor of 5 relative to other edges (presented with the shades of blue), for a better visibility. The numbers under the genera names indicate genes transferability $\gamma_{i}$. The edge between *Enterobacter* and *Klebsiella* exemplifies the assumption of the model that the HGT rate between two genera is given by the product of their tranferabilities, $\rho_{ij}=\gamma_{i}\cdot \gamma_{j}$. In addition to the fitted values of $\gamma_{i}$ presented on this plot, the fitted direct mobilome size is $L_{d}≃10^{5}$ and the size of the mobilome that goes through the hub is fitted to $L_{h}≃2.2\cdot 10^{4}$.

Consider a network hub (with index i=0) that is connected to a set *s* with *n* genera, labeled as i=1,…,n, with rates $\rho_{0i}=\gamma_{0}\gamma_{i}$ (see Fig. 3). In Section S.1.5 we show that the hub contribution to the MLD in the limit r→∞ is given by


(5) $$m_{s}^{h}(r)=2L_{h}\frac{n(n+1)}{r^{n+2}}\prod_{i=1}^{n}\rho_{0i}=2L_{h}\gamma_{0}^{n}\frac{n(n+1)}{r^{n+2}}\prod_{i=1}^{n}\gamma_{i},$$

where $L_{h}$ is the size of the mobilome that is exchanged via the hub. The scaling law of this hub-mediated long matches asymptotics is


(6) $$m_{s}^{h}(r)∼\frac{1}{r^{\alpha }}.$$

where α=n+2=|s|+2. One can also consider the hub as a common source, such that $\rho_{0i}=\gamma_{0}\gamma_{i}$, but $\rho_{i0}=0$ for each *i*. In this case the MLD is the same as in Equation (5), without the factor 2. In the following, we will assume that the rate from the hub to a genus is equal to the rate in the opposite direction. However, the same fits of the empirical MLDs can be obtained with a common source (such that the rates to the hub are all zero), just replacing $L_{h}$ by $2L_{h}$. In Sections S.1.5 and S.1.6 we provide a detailed calculation for MLD for these two cases.

#### The Complete MLD

The complete MLD is the sum of the contribution from direct transfers and from the indirect transfers that occur via the hub


(7) $$m_{s}(r)=\underset{\propto 1/r^{|s|+1}}{\underbrace{m_{s}^{d}(r)}}+\underset{\propto 1/r^{|s|+2}}{\underbrace{m_{s}^{h}(r)}}.$$

Therefore, the observed power-law exponent *α* of the empirical MLD of a given set of genera reflects the dominating HGT pathway connecting the genera in the set.

#### Number of Parameters of the Model

For a HGT network with *n* taxa we have overall n(n−1) rates $\rho_{ij}$. However, since we assume that $\rho_{ij}=\gamma_{i}\gamma_{j}$ is a rank-1 symmetric matrix, the number of free parameters is the number of n+1 transferabilities, $\gamma_{i}$, including the hub. In addition we need to model the length of the mobilome, differentiating two set of genes: genes that are transferred directly and constitute the “direct mobilome” of size $L_{d}$ and the set of genes that are transferred via the hub and constitute the “indirect mobilome” of size $L_{h}$. In total we have n+3 free parameters. The number of MLD functions that we fit is given by ∑k=2n(nk)=2n−n−1. For our case of n=8 genera, our model have 11 free parameters to fit 247 MLDs.

## Results

Having established the theoretical framework, we now evaluate it against empirical match-length distributions. Our model predicts that the MLD of an *n*-taxa set reflects the evolutionary history of HGT events. If HGTs are rare, the MLD will consist in a combination of several exponential distributions. If, however, HGTs are more frequent, the MLDs will be a combination of many exponentials and form a power-law:


(8) $$m_{n}(r)∼\frac{1}{r^{\alpha_{n}}},$$

with $\alpha_{n}=n+1$ if most of the matches are coming from the transfers within the set and $\alpha_{n}=n+2$ if indirect transfers dominate. Finally, the prefactor of the power-law depends on the HGT rates between the genera. In this Section we test these theoretical predictions on empirical data and infer the effective HGT rates.

**Fig. 4.**
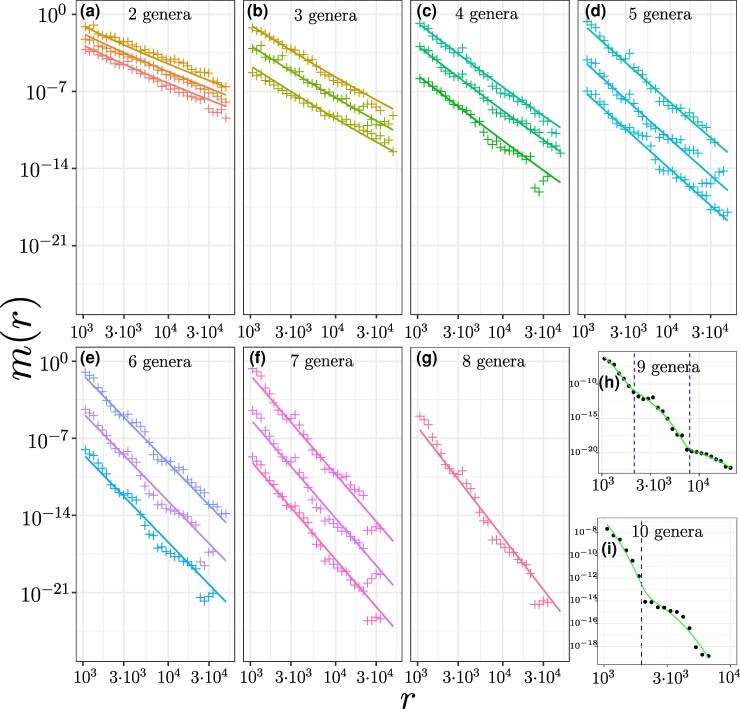
a–g) MLDs of three selected comparisons of pairs (n=2), trios (n=3), quartets (n=4) *etc* (see upper-right corners). Markers represent the empirical data and the lines represent the theoretical predictions. The MLDs are multiplies by constant prefactors for a better visibility, so the *y*-axes are arbitrary. For each set the empirical MLD and the theoretical one are multiplies by the same number. The number of considered sets for each *n* are shown in the lower-left corners. h) MLD of n=9 set: 8 considered genera plus *Vibrio*. The green line is fit with 3 exponential functions and the dashed lines represent the crossover between the exponential functions. i) MLD of n=10 set: all genera from h) and *Cronobacter*. The green line is fit with 2 exponential functions and the dashed line represents the crossover between the exponential functions. All MLDs are shown in Fig. S4 and more detailed in Figs. S5,S6,S7,S8,S9,S10).

We determine the 11 free parameters of the model by jointly fitting all 247 considered MLDs, yielding the network shown in Fig. 3 (see also inferred transferabilities in Fig. S3). The theoretical predictions show good agreement with the empirical distributions, supporting the validity of our framework. For n=2, the predicted exponent α=n+1=3 indicates that the MLDs are dominated by direct transfers (see Figs. 2a,b, 4a, Figs. S4a, S5, and Ref. Sheinman et al. 2021).

For n>2, however, the MLDs are largely dominated by matches exchanged through the hub. In particular, matches shared by n>3 taxa are well approximated by power laws with exponent α=n+2 (see Figs. 2, 4c–g, Figs. S4c–g, and S7–S10).

The case n=3 is more nuanced. For several MLDs we observe a clear transition between two regimes (see Figs. 2, 4c, Figs. S4c, and S6). These distributions are well described by a sum of two power laws with exponents α=n+1=4 and α=n+2=5: shorter matches are dominated by within-group HGT (α=4), whereas longer matches reflect indirect transfers via the hub (α=5). Consequently, the estimated values of *α* for n=3 scatter between 4 and 5 (see Fig. 2b).

### Matches Shared by Many Genera (n=9,10)

Another prediction of the model is that a single HGT event generates an exponential MLD, such that rare HGT events are expected to generate an MLD that looks like a sum of a few exponents. Moreover, if each exponent corresponds to a horizontal dissemination of a single DNA segment, the matches that contribute to it are expected to form a contiguous DNA segment.

To test these predictions, we analyze the matches which are shared by n=9,10 genera, adding *Vibrio* and *Cronobacter* to the considered genera (only for this analysis). Such widespread matches appear due to HGT events that manage to spread to a significant fraction of the population in many genera. Such events are rare and, as our theory predicts, result in MLD which can be well fitted by a combination of a few exponential distributions. The MLD of matches shared by n=9 genera is shown in Fig. 4h. This MLD can be well modelled by the combination of 3 recent events of spreading of DNA segments among the strains of considered genera. On Fig. 4i we present the MLD of the matches shared by 10 genera. In that case the MLD is dominated by only two HGT events and can be fitted by two exponential distributions. To further support this conclusion, we analyze the sequences shared by n=9,10 genera.

We can decompose MLDs for n=9 and n=10 into several exponential distributions. The crossing over from one exponent to the next (blue dashed lines in Fig. 4) define length classes of matches contributing to each component. According to our theory, we expect that all matches contributing to each of those exponential components originate from the same transferred region. Thus, the DNA sequences of the matches in each component should be related.

To test this hypothesis, we assembled the matches of each of these classes (see Supp. Mat. S.2). Matches in each class overlap sufficiently so that they can be assembled into long contigs. Notably, those belonging to the class with longest mean length can be assembled into a single contig. We show that such contigs have different functional annotations. Overall, those results are in good agreement with our theory, confirming the models’ assumptions.

### HGT Events Are Predominantly Plasmid-Mediated

Horizontal gene transfer (HGT) in bacteria occurs through three canonical mechanisms: conjugation, transduction, and transformation (Soucy et al. 2015). To assess which mechanism most likely underlies the HGT events detected in our analysis, we repeated the match-length analysis separately for chromosomal and plasmid sequences.

**Fig. 5.**
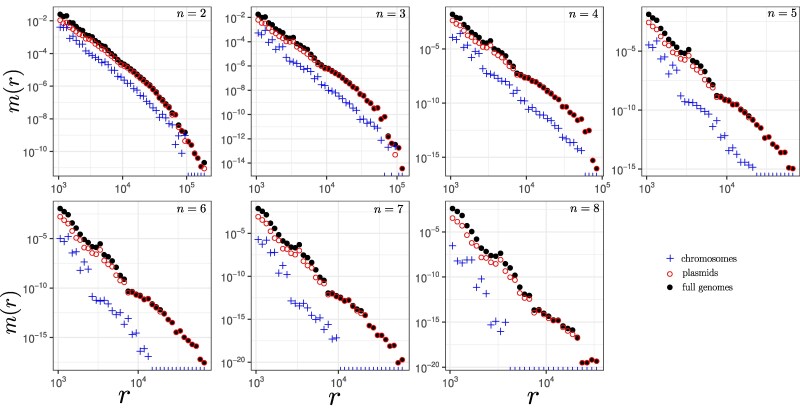
MLDs of all comparisons of pairs (n=2), trios (n=3), quartets (n=4) *etc*. Filled black circles represent all found matches, red circles represent the matches found on the plasmids, while the crosses represent the matches along the chromosomes. Note, that crosses and red circles in these plots do not have to sum up to the black dots because the black dots also include matches between the plasmids and the chromosomes.

We find that the match length distributions analyzed in this study are strongly enriched for plasmid-associated sequences (Fig. 5). This enrichment indicates that conjugation—specifically plasmid-mediated transfer—is the dominant mechanism responsible for the long exact sequence matches observed between bacterial genera. In this interpretation, the effective mobilome length inferred in our model should be understood as the total length of genes that are frequently carried on plasmids and therefore participate in recurrent horizontal exchange.

Importantly, this conclusion should be interpreted in the context of the dataset analyzed here. While plasmids are major vehicles of horizontal transfer in many bacterial systems, other mobile genetic elements can dominate gene exchange in certain taxa. In particular, integrative and conjugative elements (ICEs) are widespread and can act as major mediators of horizontal gene transfer in several bacterial groups (Cury et al. 2017; Redondo-Salvo et al. 2020; Zheng et al. 2023). Our results therefore highlight plasmids as the dominant contributors to the long exact matches detected in the present dataset, while remaining consistent with a broader diversity of transfer mechanisms across bacterial lineages.

### Abundance of Transferred Sequences is Broadly Distributed, Leading to Short Effective Mobilomes

Our model assumes a mobilome of fixed size in which all elements transfer at the same rate. Fitting the MLDs yields a direct mobilome size of $L_{d}≃\;10^{5}\;\text{bp}$ and a hub-associated mobilome size of $L_{h}≃\;2.2\times 10^{4}\;\text{bp}$, both are substantially smaller than a typical bacterial flexible genome size. To understand the origin of this unexpectedly small effective mobilome, we examined the relative abundance of horizontally transferred sequences across different genera. After clustering all identified matches into homologous groups, we quantified the relative abundance of each group. The resulting distribution (Supplemental Figs. S12 and S13) is broad and closely follows a power law with exponent −3/2. Although a theoretical model explaining this distribution is still lacking, the observed heavy tail indicates that certain sequences are transferred far more frequently than others, effectively reducing the mobilome to a small subset of highly mobile elements.

## Discussion and Summary

Horizontal gene transfer has long been recognized as a major force in bacterial evolution, but most existing approaches focus on identifying individual transfer events (see *e.g.* Ref. Evans et al. 2020) inferring the coarse-grained large-scale structure of gene flow between taxa. Recent genome-wide studies showed that long exact matches between distant bacterial genomes provide a robust signal of recent HGT (Smillie et al. 2011; Evans et al. 2020; Groussin et al. 2021; Sheinman et al. 2021; Dmitrijeva et al. 2024; Sheinman et al. 2024), while plasmid-centered analyses revealed that bacterial gene exchange is organized in complex networks rather than isolated pairwise interactions (Redondo-Salvo et al. 2020; Shapiro et al. 2023). Our work builds on these advances by showing that the full match length distribution (MLD), rather than only the presence of long matches, contains information about the topology of the underlying HGT network and about the role of indirect transfer pathways. In particular, we show that the shapes of the MLDs are well explained by a continuous process of gene flow between bacterial genera. While only pairwise HGT rates are sufficient to account for pairwise MLDs, we show that ignoring indirect HGTs would fail to successfully model the gene exchange processes occurring between bacterial genera. In contrast, our network-based approach captures emergent properties that arise from indirect HGTs via network hubs.

The exponent of the power-law, *α*, serves as a highly informative fitting parameter. Our theoretical analysis predicts that *α* should take integer values, a prediction that is well supported by empirical observations. Furthermore, *α* encapsulates information about the underlying topology of HGT network. In particular, we show that for a set of *n* interacting genera, the exponent equals α=n+1 when gene transfers occur predominantly within the set, and increases to α=n+2 when one or more external hubs act as transfer mediators or as shared sources of genes for the genera in the set.

A key conceptual result of our model is that each genus can be characterized by a scalar quantity we term its *transferability*, which reflects its intrinsic tendency to donate and receive genes. The direct HGT rate between any two genera is then given by the product of their transferabilities. The inferred transferabilities are well correlated with both the average number of plasmids per genome and the fraction of the genome shared with the other considered genera (see Figs. S14, S1, S15). This naturally explains the observed heterogeneities in gene flow and provides a minimal parameterization for modeling large-scale exchange networks. It would be interesting to relate this quantity to other measures of genome fluidity proposed in the literature, such as the pangenome openness parameter inferred from Heaps’ law fits to pangenome rarefaction curves, which capture complementary aspects of gene gain and exchange dynamics (Tettelin et al. 2008). However, such a comparison is currently limited by the availability and reliability of these estimates, as transferabilities have so far been inferred only for a small number of genera within a single family, while pangenome openness parameters have not been consistently estimated across a broad and comparable set of taxa (Costa et al. 2020). Naturally, the inferred transferability represents a coarse-grained property of a genus and may vary substantially within a genus depending on strain-specific factors, ecological context, and the particular genetic elements being transferred. Inferring a network with finer taxonomic resolution would therefore be desirable but is currently challenging due to limited genomic sampling. For the same reason, we were not able to repeat the analysis for another bacterial family, as we could not identify a family containing multiple genera with sufficiently dense genome sequencing to obtain reliable statistics.

The model predicts the presence of a high-transferability hub within the HGT network. This hub can be interpreted in several ways: it may correspond to one or a few taxa with exceptionally high transferability that act as sources of gene flow to the genera. Alternatively—and more plausibly—it may represent the collective effect of many low-transferability organisms that mediate indirect transfers between other genera. Our framework demonstrates that such an effective hub is required to reproduce the observed MLD patterns in real genomic data and provides a quantitative measure of its influence. Although the direct identification of these hub organisms is currently limited by the incomplete coverage of existing genome databases, it may become feasible with broader and more systematic genomic sampling. In this sense, our framework complements previous efforts to chart the routes of bacterial gene exchange from mobile elements themselves. Plasmidome-wide analyses have shown that plasmids form structured transfer networks across taxa (Redondo-Salvo et al. 2020; Shapiro et al. 2023), and studies of conjugative elements have likewise emphasized that HGT often occurs through highly connected vehicles rather than through isolated donor-recipient pairs (Johnson and Grossman 2015; Colombi et al. 2021; Zheng et al. 2023). Our results extend this picture by showing that indirect transfers leave a quantitative signature in the MLD exponent: pairwise rates alone can reproduce pairwise sharing statistics only incompletely, whereas network-mediated exchange through hubs naturally explains the observed higher-order patterns.

The MLD-based approach developed here is alignment-free, computationally efficient, and robust to varying degrees of sequence divergence. These properties make it particularly well-suited for analyzing diverse microbial genomes and metagenomic datasets. Unlike traditional methods that require multiple sequence alignments or phylogenetic reconstruction, our method directly leverages raw sequence similarity patterns. However, in this study we consider only the network of bacterial taxa (genera). We ignore any ecological, genetic, and other important differences between strains belonging to the same genus. Therefore, the HGT rates are effective, in the sense that they are averages over many strain-specific rates. Although we have focused on bacterial genera, the framework we propose is could be applied to other systems where horizontal transfer occurs, like microbial communities, virus-host interactions, and synthetic biology constructs.

In sum, we present a quantitative, predictive framework that connects genomic signatures to evolutionary processes on HGT networks. By linking match length statistics to network topology, our approach enables principled inference of microbial evolutionary web of life from large-scale sequencing data. This work lays the foundation for a broader understanding of genome evolution in the context of structured gene flow.

## Methods

### Finding Maximal Exact Matches

To find the maximal exact matches shared by genera pairs (sets with n=2) we used bfmem software (Liu et al. 2019). To find matches for sets with n>2 we intersect the matches found for pairs. The script to do so can be found in the github repository (Sheinman et al. 2025).

### Calculating and Fitting MLDs

For the MLDs we used logarithmic binning with 20 points per decade. Namely, our bins edges are $(1,000-0.5)\cdot 10^{0.05k}$ where *k* is an integer. In the plots, the centers of the bins are the geometric means of their edges. Within each bin we count the number of matches and normalize it by the size of the bin. In addition, we normalize the MLD by the total number of alignments we perform for the considered set. If we analyze set *s* of size |s|=n with $k_{1},k_{2},\ldots ,k_{n}$ genomes in the database, we divide the total MLD by $k_{1}k_{2}\cdots k_{n}$.

To fit the parameters of the model we minimize the mean squared log-difference between the empirical and the predicted MLDs. The mean is 15% trimmed: to make it more robust, the worst 15% of the bins for each set are not included in the mean. The minimization was performed in two steps: global GA (Scrucca 2013) and then L-BFGS-B (Zhu et al. 1997) optimization using the optimParallel package (Gerber 2021) in R.

The estimate of the power-law exponent *α* for each set is obtained using the method-of-moments estimator (Quandt 1964). All relevant scripts can be found in github repository (Sheinman et al. 2025).

## Supplementary Material

evag099_Supplementary_Data

## Data Availability

We used seven genera from the *Enterobacteriaceae* family and one close outgroup: the *Serratia* genus. To see how the power law breaks into a mixture of exponentials, we added the genus *Cronobacter*, which has a small number (46) of sequenced genomes in the database, and the phylogenetically distant genus *Vibrio*. We used only complete genome-level assemblies in the RefSeq (O’Leary et al. 2016) database via NCBI (Sayers et al. 2023). The family and the genera included in the analysis were selected primarily based on genome availability and sampling depth, ensuring sufficient numbers of genomes to obtain reliable statistics of exact matches. To mitigate sampling biases we removed samples obtained from large multi-isolate projects, as they are indicated in the NCBI site. The total number of analyzed genomes for each genus is given in Table 1. The list of genomes with their accession numbers and the bash script to download the data is deposited on github (Sheinman et al. 2025). Number of genomes analyzed for each bacterial genus included in the study.

## References

[evag099-B1] Arndt PF . Sequential and continuous time stick-breaking. J Stat Mech. 2019:2019:064003. 10.1088/1742-5468/ab1dd8.

[evag099-B2] Bapteste E et al Prokaryotic evolution and the tree of life are two different things. Biol Direct. 2009:4:34. 10.1186/1745-6150-4-34. 19788731 PMC2761302

[evag099-B3] Battistuzzi FU, Feijao A, Hedges SB. A genomic timescale of prokaryote evolution: insights into the origin of methanogenesis, phototrophy, and the colonization of land. BMC Evol Biol. 2004:4:1–14. 10.1186/1471-2148-4-1. 15535883 PMC533871

[evag099-B4] Carr VR, Shkoporov A, Hill C, Mullany P, Moyes DL. Probing the mobilome: discoveries in the dynamic microbiome. Trends Microbiol. 2021:29:158–170. 10.1016/j.tim.2020.05.003. 32448763

[evag099-B5] Colombi E et al Comparative analysis of integrative and conjugative mobile genetic elements in the genus mesorhizobium. Microb Genom. 2021:7:000657. 10.1099/mgen.0.000657. 34605762 PMC8627217

[evag099-B6] Cordero OX, Polz MF. Explaining microbial genomic diversity in light of evolutionary ecology. Nat Rev Microbiol. 2014:12:263–273. 10.1038/nrmicro3218. 24590245

[evag099-B7] Corel E, Lopez P, Méheust R, Bapteste E. Network-thinking: graphs to analyze microbial complexity and evolution. Trends Microbiol. 2016:24:224–237. 10.1016/j.tim.2015.12.003. 26774999 PMC4766943

[evag099-B8] Costa SS, Guimarães LC, Silva A, Soares SC, Baraúna RA. First steps in the analysis of prokaryotic pan-genomes. Bioinform Biol Insights. 2020:14:1177932220938064. 10.1177/1177932220938064. 32843837 PMC7418249

[evag099-B9] Cury J, Touchon M, Rocha EP. Integrative and conjugative elements and their hosts: composition, distribution and organization. Nucleic Acids Res. 2017:45:8943–8956. 10.1093/nar/gkx607. 28911112 PMC5587801

[evag099-B10] Dagan T, Artzy-Randrup Y, Martin W. Modular networks and cumulative impact of lateral transfer in prokaryote genome evolution. Proc Natl Acad Sci U S A. 2008:105:10039–10044. 10.1073/pnas.0800679105. 18632554 PMC2474566

[evag099-B11] Darwin C . The origin of species by means of natural selection, or, the preservation of favoured races in the struggle for life. Books, Incorporated, Pub; 1859. PMC5184128 30164232

[evag099-B12] Didelot X, Walker AS, Peto TE, Crook DW, Wilson DJ. Within-host evolution of bacterial pathogens. Nat Rev Microbiol. 2016:14:150–162. 10.1038/nrmicro.2015.13. 26806595 PMC5053366

[evag099-B13] Dixit PD, Pang TY, Studier FW, Maslov S. Recombinant transfer in the basic genome of escherichia coli. Proc Natl Acad Sci U S A. 2015:112:9070–9075. 10.1073/pnas.1510839112. 26153419 PMC4517234

[evag099-B14] Dmitrijeva M et al A global survey of prokaryotic genomes reveals the eco-evolutionary pressures driving horizontal gene transfer. Nat Ecol Evol. 2024:8:986–998. 10.1038/s41559-024-02357-0. 38443606 PMC11090817

[evag099-B15] Doolittle WF . Phylogenetic classification and the universal tree. Science. 1999:284:2124–2128. 10.1126/science.284.5423.2124. 10381871

[evag099-B16] Drake JW . A constant rate of spontaneous mutation in DNA-based microbes. Proc Natl Acad Sci U S A. 1991:88:7160–7164. 10.1073/pnas.88.16.7160. 1831267 PMC52253

[evag099-B17] Duchêne S, et al Genome-scale rates of evolutionary change in bacteria. Microb Genom. 2016:2:e000094. 10.1099/mgen.0.000094. 28348834 PMC5320706

[evag099-B18] Evans DR et al Systematic detection of horizontal gene transfer across genera among multidrug-resistant bacteria in a single hospital. Elife. 2020:9:e53886. 10.7554/eLife.53886. 32285801 PMC7156236

[evag099-B19] Gerber F, Furrer R. optimparallel: an R package providing a parallel version of the L-BFGS-B optimization method. R J. 2019:11:352–358. 10.32614/RJ-2019-030.

[evag099-B20] Groussin M et al Elevated rates of horizontal gene transfer in the industrialized human microbiome. Cell. 2021:184:2053–2067. 10.1016/j.cell.2021.02.052. 33794144

[evag099-B21] Harris K, Nielsen R. Inferring demographic history from a spectrum of shared haplotype lengths. PLoS Genet. 2013:9:e1003521. 10.1371/journal.pgen.1003521. 23754952 PMC3675002

[evag099-B22] Johnson CM, Grossman AD. Integrative and conjugative elements (ICEs): what they do and how they work. Annu Rev Genet. 2015:49:577–601. 10.1146/annurev-genet-112414-055018. 26473380 PMC5180612

[evag099-B23] Khaledian E, Brayton KA, Broschat SL. A systematic approach to bacterial phylogeny using order level sampling and identification of HGT using network science. Microorganisms. 2020:8:312. 10.3390/microorganisms8020312. 32102454 PMC7074868

[evag099-B24] Kumar S, Stecher G, Suleski M, Hedges SB. Timetree: a resource for timelines, timetrees, and divergence times. Mol Biol Evol. 2017:34:1812–1819. 10.1093/molbev/msx116. 28387841

[evag099-B25] Kunin V, Goldovsky L, Darzentas N, Ouzounis CA. The net of life: reconstructing the microbial phylogenetic network. Genome Res. 2005:15:954–959. 10.1101/gr.3666505. 15965028 PMC1172039

[evag099-B26] Li C, Chen J, Li SC. Understanding horizontal gene transfer network in human gut microbiota. Gut Pathog. 2020:12:33. 10.1186/s13099-020-00370-9. 32670414 PMC7346641

[evag099-B27] Liu Y, Zhang LY, Li J. Fast detection of maximal exact matches via fixed sampling of query k-mers and bloom filtering of index k-mers. Bioinformatics. 2019:35:4560–4567. 10.1093/bioinformatics/btz273. 30994891

[evag099-B28] López Sánchez A, Lafond M. Predicting horizontal gene transfers with perfect transfer networks. Algorithms Mol Biol. 2024:19:6. 10.1186/s13015-023-00242-2. 38321476 PMC10848447

[evag099-B29] Massip F, Arndt PF. Neutral evolution of duplicated DNA: an evolutionary stick-breaking process causes scale-invariant behavior. Phys Rev Lett. 2013:110:148101. 10.1103/PhysRevLett.110.148101. 25167038

[evag099-B30] Massip F, Sheinman M, Schbath S, Arndt PF. How evolution of genomes is reflected in exact DNA sequence match statistics. Mol Biol Evol. 2015:32:524–535. 10.1093/molbev/msu313. 25398628 PMC4298173

[evag099-B31] Massip F, Sheinman M, Schbath S, Arndt PF. Comparing the statistical fate of paralogous and orthologous sequences. Genetics. 2016:204:475 482. 10.1534/genetics.116.193912. 27474728 PMC5068840

[evag099-B32] Novichkov PS et al Genome-wide molecular clock and horizontal gene transfer in bacterial evolution. J Bacteriol. 2004:186:6575–6585. 10.1128/JB.186.19.6575-6585.2004. 15375139 PMC516599

[evag099-B33] Ochman H, Elwyn S, Moran NA. Calibrating bacterial evolution. Proc Natl Acad Sci U S A. 1999:96:12638–12643. 10.1073/pnas.96.22.12638. 10535975 PMC23026

[evag099-B34] Ochman H, Lawrence JG, Groisman EA. Lateral gene transfer and the nature of bacterial innovation. Nature. 2000:405:299–304. 10.1038/35012500. 10830951

[evag099-B35] O’Leary NA et al Reference sequence (RefSeq) database at NCBI: current status, taxonomic expansion, and functional annotation. Nucleic Acids Res. 2016:44:D733–D745. 10.1093/nar/gkv1189. 26553804 PMC4702849

[evag099-B36] Partridge SR, Kwong SM, Firth N, Jensen SO. Mobile genetic elements associated with antimicrobial resistance. Clin Microbiol Rev. 2018:31:10–1128. 10.1128/CMR.00088-17. PMC6148190 30068738

[evag099-B37] Quandt RE . Old and mew methods of estimation and the Pareto distribution. Metrika. 1964:10:55–82. 10.1007/BF02613419.

[evag099-B38] Ravenhall M, Škunca N, Lassalle F, Dessimoz C. Inferring horizontal gene transfer. PLoS Comput Biol. 2015:11:e1004095. 10.1371/journal.pcbi.1004095. 26020646 PMC4462595

[evag099-B39] Redondo-Salvo S et al Pathways for horizontal gene transfer in bacteria revealed by a global map of their plasmids. Nat Commun. 2020:11:3602. 10.1038/s41467-020-17278-2. 32681114 PMC7367871

[evag099-B40] Sakoparnig T, Field C, van Nimwegen E. Whole genome phylogenies reflect the distributions of recombination rates for many bacterial species. Elife. 2021:10:e65366. 10.7554/eLife.65366. 33416498 PMC7884076

[evag099-B41] Sayers EW et al Database resources of the national center for biotechnology information in 2023. Nucleic Acids Res. 2023:51:D29–D38. 10.1093/nar/gkac1032. 36370100 PMC9825438

[evag099-B42] Scrucca L . Ga: a package for genetic algorithms in R. J Stat Softw. 2013:53:1–37. 10.18637/jss.v053.i04.

[evag099-B43] Shapiro JT et al Multilayer networks of plasmid genetic similarity reveal potential pathways of gene transmission. ISME J. 2023:17:649–659. 10.1038/s41396-023-01373-5. 36759552 PMC10119158

[evag099-B44] Sheinman M et al Identical sequences found in distant genomes reveal frequent horizontal transfer across the bacterial domain. Elife. 2021:10:e62719. 10.7554/eLife.62719. 34121661 PMC8270642

[evag099-B45] Sheinman M, Arndt PF, Massip F. Modeling the mosaic structure of bacterial genomes to infer their evolutionary history. Proc Natl Acad Sci U S A. 2024:121:e2313367121. 10.1073/pnas.2313367121. 38517978 PMC10990148

[evag099-B46] Sheinman M, Stentella T, Etheimer P, Massip F, Arndt PF. https://github.com/mishashe/networkHGT. 2025. 10.1093/gbe/evag099 PMC13148533 41999584

[evag099-B47] Siefert JL . Defining the mobilome. In: Gogarten, MB, Gogarten, JP, Olendzenski, L.C, editors. Horizontal Gene Transfer. Humana Press; 2009. p. 13.

[evag099-B48] Smillie CS et al Ecology drives a global network of gene exchange connecting the human microbiome. Nature. 2011:480:241–244. 10.1038/nature10571. 22037308

[evag099-B49] Šorfová P, Škeříková A, Hypša V. An effect of 16s rRNA intercistronic variability on coevolutionary analysis in symbiotic bacteria: molecular phylogeny of arsenophonus triatominarum. Syst Appl Microbiol. 2008:31:88–100. 10.1016/j.syapm.2008.02.004. 18485654

[evag099-B50] Soucy SM, Huang J, Gogarten JP. Horizontal gene transfer: building the web of life. Nat Rev Genet. 2015:16:472–482. 10.1038/nrg3962. 26184597

[evag099-B51] Tettelin H, Riley D, Cattuto C, Medini D. Comparative genomics: the bacterial pan-genome. Curr Opin Microbiol. 2008:11:472–477. 10.1016/j.mib.2008.09.006. 19086349

[evag099-B52] Treangen TJ, Rocha EP. Horizontal transfer, not duplication, drives the expansion of protein families in prokaryotes. PLoS Genet. 2011:7:e1001284. 10.1371/journal.pgen.1001284. 21298028 PMC3029252

[evag099-B53] Zhao S et al Adaptive evolution within gut microbiomes of healthy people. Cell Host Microbe. 2019:25:656–667. 10.1016/j.chom.2019.03.007. 31028005 PMC6749991

[evag099-B54] Zheng Q, Li L, Yin X, Che Y, Zhang T. Is ICE hot? a genomic comparative study reveals integrative and conjugative elements as “hot” vectors for the dissemination of antibiotic resistance genes. mSystems. 2023:8:e0017823. 10.1128/msystems.00178-23. 38032189 PMC10734551

[evag099-B55] Zhu C, Byrd RH, Lu P, Nocedal J. Algorithm 778: L-bfgs-b: fortran subroutines for large-scale bound-constrained optimization. ACM Trans Math Softw (TOMS). 1997:23:550–560. 10.1145/279232.279236.

[evag099-B56] Ziff RM, McGrady E. The kinetics of cluster fragmentation and depolymerisation. J Phys A Math Gen. 1985:18:3027–3037. 10.1088/0305-4470/18/15/026.

[evag099-B57] Zuckerkandl E, Pauling L. Evolutionary divergence and convergence in proteins. In: Evolving genes and proteins. Elsevier; 1965. p. 97–166.

