# Supplementary material for: Reconstructing the Network of Horizontal Gene Exchange in Bacteria to Differentiate Direct and Indirect Transfers
Source: Genome Biol Evol. 2026 Apr 18;18(5):evag099. doi: 10.1093/gbe/evag099 (PMC13148533; doi:10.1093/gbe/evag099)
Supplement: evag099_Supplementary_Data [file evag099_supplementary_data.zip › supplementary.pdf]

Supplemental Results

Reconstructing the network of horizontal gene exchanges in bacteria to differentiate direct and indirect transfers

Michael Sheinman[1,2], Tommaso Stentella[3], Paul Etheimer[4,5,6], Florian Massip[4,5,6], Peter F. Arndt[3]

[1]Department of Physics of Complex Systems, Weizmann Institute of Science, Rehovot, Israel
[2]Institute for Advanced Studies, Sevastopol State University, Sevastopol, Russia
[3]Max Planck Institute for Molecular Genetics, Berlin, Germany
[4]Centre for Computational Biology (CBIO), Mines Paris, PSL University, Paris, France
[5]Institut Curie, PSL University, Paris, France
[6]INSERM, U900, Paris, France

S.1 General equation and its analytic solution

In this section we present the model and derive in more detail analytical results for the expected match length distribution (MLD) for a given HGT network. As network nodes we use bacterial taxa (we use genera in this article, but in this section we keep it more general), while the edges represent the effective rates of HGT between the taxa pairs. Effective rates take into account that the transferred gene is not present in 100% of the genomes of the donor taxon and is not transferred to 100% of genomes of the recipient taxon. We assume that all transfers are Poisson processes with different rates and focusing on long matches shared by distant taxa, thereby neglecting the contribution of conservation to the exact sequence matches. Throughout the paper we define time units such that the mutation rate $\mu = 1$, such that all inferred effective HGT rates are given in units of a typical bacterial mutation rate μ.

We consider network of taxa exchanging genetic material from a mobilome of length L. The exchange rate from taxon i to taxon j is denoted by ρ_{ij}. We obtain the analytic solution for a general asymmetric matrix, but to fit the model we assume that every taxon i is characterized by its transferability γ_i, such that the transfer matrix is symmetric and rank-1, given by $\rho_{ij} = \gamma_i \gamma_j$.

To simplify the notation we define $\rho_{ii} = 0$. For a set of distinct taxa $s = \{i, j, k, ...\}$ with $i < j < k < ...$, we define $P_s(\tau)d\tau$ as the expected fraction of the mobilome for which the total length of the phylogenetic tree of the set s is in the interval $[\tau, \tau + d\tau]$. For a trivial set of a single taxon, obviously, $P_{\{i\}}(\tau) = \delta(\tau)\ \forall i$. For completeness we solve separately the case with the additive HGT and replacing one, although the asymptotic behaviour is the same in both cases and for the regime that is relevant for our study both scenarios are practically indistinguishable.

S.1.1 How to relate $P_s(\tau)$ to genomic data

Before we proceed to solve the model, we discuss here how to validate our model using empirical data: how to relate calculated $P_s(\tau)$ to an empirical quantity. In principle, using the molecular clock assumption, evolutionary time divergence τ between two DNA loci is related to the fraction of mismatches. However, as stated above, our model results in mosaic structure of the genome, such that different loci possess different time divergences. Thus, a simple molecular clock with a single divergence time cannot be used. Instead we have a combination of clocks in different loci—each with a different divergence time. Furthermore, for real bacterial genomes we don't know where one locus ends and another one starts, so one cannot calculate $P_{ij}(\tau)$ directly from two bacterial genomes to compare it to the one that we derive below using our model. A method to circumvent this problem was presented and used in Refs. (Harris and Nielsen, 2013; Massip and Arndt, 2013; Massip et al., 2015, 2016; Sheinman et al., 2021). The main idea is to use statistical properties of the exact sequence matches between the homologous sequences of a pair instead of the statistical properties of time divergence. One can show (Arndt, 2019), using the result derived in (Ziff and McGrady, 1985) that the molecular clock instead of fraction of mismatches can be formulated in terms of the distribution of exact matches lengths r. Namely, for a given divergence

τ between two loci of length K the expected number of their exact sequence matches of length $r \ll K$ is given by

$$m(r) = K\tau^2 e^{-\tau r}. \tag{S1}$$

Therefore, if the divergence probability between strains i and j is $P_{ij}(\tau)$ the exact match length distribution of i and j genomes of length L (with $\simeq L/K$ loci) is given by

$$m_{ij}(r) = L\int_0^\infty \tau^2 e^{-r\tau} P_{ij}(\tau) d\tau = L\frac{d^2 \widetilde{P}_{ij}(r)}{dr^2} \tag{S2}$$

where $\widetilde{P}_{ij}(r) = \mathscr{L}\{P_{ij}\}(r) = \int_0^\infty e^{-r\tau} P_{ij}(\tau) d\tau$ is the Laplace transform of $P_{ij}(\tau)$. One can see that length distribution of exact sequence matches between two sequences is related to the Laplace transform of their time divergence distribution. This is why in the following we analytically calculate not only $P_{ij}(\tau)$ but also $m_{ij}(r)$ because the last quantity can be relatively easily obtained from the empirical data, in contrast to $P_{ij}(\tau)$.

In the following we split our analysis into two different types of HGT: additive and replacing (Choi et al., 2012; Kloub et al., 2024). The asymptotic, large r results will be identical in both cases.

S.1.2 Additive HGT

We consider a set of taxa s exchanging genetic material from a mobilome of length L. To simplify the notation we define $\rho_{ii} = 0$ and $P_{\{i\}}(\tau) = \delta(\tau)\ \forall i$. So far we implicitly referred to $P_s(\tau, t)$ without the time dependence t, i.e. we referred to the steady state of the tree length distribution. We are interested in the steady state, as it does not depend on the sampling time. Here we consider the scenario when the HGT event does not replace its homolog in the recipient but simply adds the transferred sequence to the recipient's genome. In this case the time evolution of $P_s(\tau, t)$ can be written in a recurrent form. The recurrent form is expressed in term of P of one of the two following sets: $\{i, s\backslash j\}$ sets where taxon $j \in s$ from the set s is replaced by taxon $i \notin s$ (i.e. incoming gene), and the subsets of the kind $s\backslash j$ which are obtained removing taxon $j \in s$ from s.

$$\frac{\partial P_s(\tau,t)}{\partial t} = \sum_{i\notin s, j\in s} \rho_{ij} P_{\{i,s\backslash j\}}(\tau,t) + \sum_{i\in s, j\in s} \rho_{ij} P_{s\backslash j}(\tau,t) - |s|\frac{\partial P_s(\tau,t)}{\partial \tau}. \tag{S3}$$

The first term represents the transfers from taxa $i \notin s$ outside of s, while the second term represents transfers within the set. The third term represents accumulation of the total evolutionary distance of the set s with $|s|$ members. Because the HGT is additive, the sink term in the equation is absent.

Defining the matrix R^{a} in the sets space (superscript a stands for "additive"),

$$-R^{\mathrm{a}} P_s = \sum_{i\notin s, j\in s} \rho_{ij} P_{\{i,s\backslash j\}}, \tag{S4}$$

in the Laplace r-space, the steady state is given by

$$\widetilde{P}_s(r) = (|s| r \mathbb{1} + R^{\mathrm{a}})^{-1} \sum_{i\in s, j\in s} \rho_{ij} \widetilde{P}_{s\backslash j}(r). \tag{S5}$$

Therefore, one can calculate $\widetilde{P}_s(r)$ of all sets s with n members using only information about sets with $n-1$ members. Starting from trivial sets with one member $\widetilde{P}_{\{i\}}(r) = 1$ one can iteratively calculate $\widetilde{P}_s(r)$ for all subsets s of the taxa network using Eq. (S5). Then, defining time such that the mutation rate is $\mu = 1$, one can get the expected MLD of set s using (S2):

$$m_s(r) = L\int_0^\infty P_s(\tau)\tau^2 e^{-r\tau} d\tau = L\widetilde{P}_s''(r). \tag{S6}$$

S.1.3 Replacing HGT

In the replacing scenario for a set of distinct taxa $s = \{i, j, k, ...\}$ with $i < j < k < ...$ the evolution of $P_s(\tau, t)$ is given by

$$\frac{\partial P_s(\tau,t)}{\partial t} = \sum_{i\notin s, j\in s} \rho_{ij}\left(P_{\{i,s\backslash j\}}(\tau,t) - P_s(\tau,t)\right) + \sum_{i\in s, j\in s} \rho_{ij}\left(P_{s\backslash j}(\tau,t) - P_s(\tau,t)\right) - |s|\frac{\partial P_s(\tau,t)}{\partial \tau}. \tag{S7}$$

In the steady state one can write

$$|s|\frac{\partial P_s}{\partial \tau} = \sum_{i\notin s, j\in s} \rho_{ij}\left(P_{\{i,s\backslash j\}} - P_s\right) + \sum_{i\in s, j\in s} \rho_{ij}\left(P_{s\backslash j} - P_s\right) \tag{S8}$$

Defining the matrix R^{r} in the sets space (superscript r stands for "replacing")

$$-R^{\mathrm{r}} P_s = \sum_{i\notin s, j\in s} \rho_{ij}\left(P_{\{i,s\backslash j\}} - P_s\right) - \sum_{i\in s, j\in s} \rho_{ij} P_s \tag{S9}$$

in the Laplace r-space

$$|s|r\widetilde{P}_s - |s|P_s(0) = \sum_{i\notin s, j\in s} \rho_{ij}\left(\widetilde{P}_{\{i,s\backslash j\}} - \widetilde{P}_s\right) + \sum_{i\in s, j\in s} \rho_{ij}\left(\widetilde{P}_{s\backslash j} - \widetilde{P}_s\right) = -R^{\mathrm{r}}\widetilde{P}_s + \sum_{i\in s, j\in s} \rho_{ij}\widetilde{P}_{s\backslash j} \tag{S10}$$

one can simplify to

$$|s|r\widetilde{P}_s - |s|P_s(0) = -R^{\mathrm{r}}\widetilde{P}_s + \sum_{i\in s, j\in s} \rho_{ij}\widetilde{P}_{s\backslash j}. \tag{S11}$$

Demanding $\widetilde{P}_s(0) = 1$ we get

$$-|s|P_s(0) = -R^{\mathrm{r}} \cdot \mathbf{1} + \sum_{i\in s, j\in s} \rho_{ij} = 0 \tag{S12}$$

such that

$$\widetilde{P}_s = \left(|s|r\mathbb{1} + R^{\mathrm{r}}\right)^{-1}\left[\sum_{i\in s, j\in s} \rho_{ij}\left(\widetilde{P}_{s\backslash j} - 1\right) + R^{\mathrm{r}} \cdot \mathbf{1}\right] = \left(|s|r\mathbb{1} + R^{\mathrm{r}}\right)^{-1}\sum_{i\in s, j\in s} \rho_{ij}\widetilde{P}_{s\backslash j}. \tag{S13}$$

Thus, the recursive equation for $\widetilde{P}_s$ in the replacing HGT case is the same as for the additive HGT case, but R^{r} matrix (S9) is different from the R^{a} matrix for additive HGT (given in Eq. (S4)).

S.1.4 Asymptotic results for both additive and replacing HGT are the same

One can easily see that Eqs. (S5) and (S13) in the large r limit do not not depend on R^{a} or R^{r} and become identical. Below we give explicit solution in this asymptotic regime for both additive and replacing HGT scenarios, first for s of different sizes and later for a general set s.

S.1.4.1 Two taxa $n = |s| = 2$

In the limit $r \to \infty$ Eqs. (S5) and (S13) for $n = 2$ are reduced to

$$\widetilde{P}_{12}(r) = \frac{\rho_{12} + \rho_{21}}{2r} = \frac{\sum_{i,j\in\{1,2\}} \rho_{ij}}{2} \tag{S14}$$

and

$$m_{12}(r) = L\widetilde{P}''_{12}(r) = L\frac{\rho_{12} + \rho_{21}}{r^3}. \tag{S15}$$

S.1.4.2 Three taxa

In the limit $r \to \infty$ Eqs. (S5) and (S13) for $n = 3$ are reduced to

$$\widetilde{P}_{123}(r) = \sum_{i,j\in\{123\}} \rho_{ij}\frac{\sum_{k,\ell\in 123\backslash j} \rho_{k\ell}}{6r^2}. \tag{S16}$$

In the explicit form

$$m_{123}(r) = L\frac{\sum\limits_{i,j\in\{123\}} \rho_{ij} \sum\limits_{k,\ell\in 123\backslash j} \rho_{k\ell}}{r^4} = L\frac{(\rho_{12} + \rho_{21})(\rho_{23} + \rho_{31}) + (\rho_{13} + \rho_{31})(\rho_{12} + \rho_{32}) + (\rho_{13} + \rho_{21})(\rho_{23} + \rho_{32})}{r^4}. \tag{S17}$$

S.1.4.3 Four taxa

Using the same line of arguments

$$\widetilde{P}_{1234}(r) = \frac{1}{24r^3} \sum_{i,j\in\{1234\}} \rho_{ij} \sum_{k,\ell\in 1234\backslash j} \rho_{k\ell} \sum_{m,n\in 1234\backslash j\backslash \ell} \rho_{mn} \tag{S18}$$

so

$$m_{1234}(r) = \frac{L}{2r^5} \sum_{i,j\in\{1234\}} \rho_{ij} \sum_{k,\ell\in 1234\backslash j} \rho_{k\ell} \sum_{m,n\in 1234\backslash j\backslash \ell} \rho_{mn} \tag{S19}$$

S.1.4.4 Five taxa

In a network of HGT with 5 taxa the MLD tail is given by

$$m_{12345}(r) = \frac{4\cdot 5L}{2\cdot 3\cdot 4\cdot 5r^{5+1}} \sum_{i_1j_1\in\{12345\}} \rho_{i_1j_1} \sum_{i_2j_2\in 12345\backslash j_1} \rho_{i_2j_2} \sum_{i_3j_3\in 12345\backslash j_1\backslash j_2} \rho_{i_3j_3} \sum_{i_4j_4\in 12345\backslash j_1\backslash j_2\backslash j_3} \rho_{i_4j_4} \tag{S20}$$

S.1.4.5 General asymptotic result

In a s-network of HGT with $|s| = n$ taxa and asymmetric transfer rates ρ_{ij} the MLDs tails are given by Eq. (3):

$$m_s(r) = \frac{L}{(n-2)!r^{n+1}} \sum_{i_1j_1\in s} \rho_{i_1j_1} \sum_{i_2j_2\in s\backslash j_1} \rho_{i_2j_2} \cdots \sum_{i_{n-1}j_{n-1}\in s\backslash j_1\backslash j_2\cdots\backslash j_{n-2}} \rho_{i_{n-1}j_{n-1}}. \tag{S21}$$

The MLD of matches shared by the same set of genera, but embedded in a larger network will have $\frac{1}{r^{n+2}}$ correction term to this asymptotic result. In the case when there is a strong hub outside of the set, this correction term may become dominant. In the following we calculate the $\frac{1}{r^{n+2}}$ term for such a strong hub.

S.1.5 Common strong hub calculation

To calculate the contribution of a strong hub to the MLD we consider a hub which is connected to n genera. The latter have no direct connections with each other and transfer the genes only via the hub. We calculate $P_s(\tau)$, $\widetilde{P}_s(r)$, $m(r)$ in the asymptotic $\tau \to 0$, $r \to \infty$ regime. For convenience, we denote the hub with index 0 and the other genera with index $i = 1...n$.

Since the hub is connected directly to each genus i with rate ρ_{0i}, in the asymptotic regime, as we found above

$$\widetilde{P}_{0i}(r) = \frac{2\rho_{0i}}{r}. \tag{S22}$$

To find the pairwise MLDs we write

$$2r\widetilde{P}_{ij}(r) = \rho_{0i}\widetilde{P}_{0j}(r) + \rho_{0j}\widetilde{P}_{0i}(r) \tag{S23}$$

or

$$\widetilde{P}_{ij}(r) = \frac{2\rho_{0i}\rho_{0j}}{r^2}. \tag{S24}$$

To find the MLDs of 3 genera we write

$$3r\widetilde{P}_{ijk}(r) = \rho_{0i}\widetilde{P}_{0jk}(r) + \rho_{0j}\widetilde{P}_{0ik}(r) + \rho_{0k}\widetilde{P}_{0ij}(r) \tag{S25}$$

or

$$\widetilde{P}_{ijk}(r) = \frac{2\rho_{0i}\rho_{0j}\rho_{0k}}{r^3}. \tag{S26}$$

In the special case of $\rho_{ij} = \gamma_i\gamma_j$ we get

$$\widetilde{P}_s(r) = \frac{2\gamma_0^n}{r^n}\prod_{i=1}^{n}\gamma_i. \tag{S27}$$

and match length distribution in the limit $r \to \infty$ is given by Eq. (5)

$$m_s(r) = \frac{2n(n+1)L\gamma_0^n}{r^{n+2}}\prod_{i=1}^{n}\gamma_i. \tag{S28}$$

S.1.6 Common source calculation

In the calculation above we considered the edges between the hub and the other n nodes of the network as symmetric. Here we analyze the case when the HGT can occur only from the hub to an other node, but not in the opposite direction. In this case the hub acts as a common source of genes. We show now that in the asymptotic regime a common source can be viewed as a simple hub with transferability divided by $2^{1/n}$. Consider a common hub (with index $i = 0$) which act as a gene donor to $i = 1, \ldots, n$ genera with rates $\rho_{0i} = \gamma_0\gamma_i$. Let's say that the HGT from the hub to the genera occurred times $\tau_1, \tau_2, \ldots, \tau_n$ ago. For τ_i the indices i indicate not the taxa, but the rank of the transfer event, such that $\tau_1 \geq \tau_2 \geq ... \geq \tau_n$. The sum of all times at which mutations could accumulate is given by

$$\tau = \tau_1 - \tau_n + \sum_{i=1}^{n} \tau_i \tag{S29}$$

and the distribution of τ in the $\tau \to 0$ limit is given by

$$P_s(\tau) = \frac{\tau^{n-1}}{n!} \prod_{i=1}^{n} \gamma_0\gamma_i. \tag{S30}$$

Thus its Laplace transform in the limit $r \to \infty$ is

$$\widetilde{P}_s(r) = \frac{1}{r^n} \prod_{i=1}^{n} \gamma_0\gamma_i \tag{S31}$$

and match length distribution in the limit $r \to \infty$ is given by

$$m_s(r) = \frac{n(n+1)L\gamma_0^n}{r^{n+2}} \prod_{i=1}^{n} \gamma_i. \tag{S32}$$

One can see that (S32) is equivalent to halved (5) or γ_0 transferability divided by $2^{1/n}$. In our study we assume that the hub is connected to other genera with symmetric edges, but, as show here, our results can be easily extended to the common source case redefining γ_0. The function form of the MLD is the same $m_s \sim r^{-(n+2)}$ in both cases. This scaling is robust to system's parameters and is qualitatively different from the $m_s \sim r^{-(n+1)}$ scaling for the system where the transfers are predominantly within the set s (see Eqs. (3,4)).

S.2 Matches shared by many $n = 9, 10$ genera

Here we analyze the matches which are shared by $n = 9, 10$ genera to test the limits of our assumptions of continuous HGT. To do so we added *Vibrio* and *Cronobacter* to the considered genera (only for this analysis) and found matches shared by genera from these augmented sets. These matches appear due to HGT events which manage to spread in significant fractions of the population in many genera. Such events are rare and, as our theory predicts, result in MLD which can be well fitted by a combination of a few exponentials.

As an instructive example consider matches shared by $n = 9$ genera: *Escherichia, Klebsiella, Salmonella, Enterobacter, Vibrio, Citrobacter, Serratia, Proteus, Raoultella.* The MLD of this comparison is shown in Fig. 4(h). One can see that the MLD can be well modelled by 3 recent events of spreading of DNA segments in the strains of considered genera. To get more insight into the details of these 3 processes we assembled separately short (1000 bp $\leq r <$ 2154 bp), medium (2154 bp $\leq r <$ 8000 bp) and long ($r \geq$ 8000 bp) matches using `metaMDBG` (Benoit et al., 2024).

Long matches Long matches are assembled to a single contig of length 19,411 bp. Functional annotation of genes along this contig (performed using `eggNOG-mapper v2` (Cantalapiedra et al., 2021)) results in TraL, TraE, TraK, TrbI, and TraV: genes, associated with pili assembly and production (Zatyka and Thomas, 1998). In addition there are cytosine-specific DNA methylase, Bacterial DNA topoisomerase I and relaxase. Blasting the contig against the RefSeq database we find thousands of plasmids and bacterial chromosomes with almost a perfect match to the contig. This means that this contig is a mobile element, widespread in many bacteria species, beyond the set we are studying in this paper.

Medium matches Medium matches assemble to 11 contigs of total length 51318 bp. The genes located at the matches are associated with fluoroquinolone, chromate, mercury and multidrug resistance, detoxification of formaldehyde, transposase, resolvase, chemotaxis and phage integrase.

Short matches Short matches assemble to 3 contigs of total length 11575 bp. The genes located at the matches are adenine-specific DNA-methyltransferase, Beta-lactamase, chemotaxis, Cupin 2 and Transposase.

Analysis of matches shared by all 10 genera (*Escherichia, Klebsiella, Salmonella, Enterobacter, Citrobacter, Serratia, Proteus, Raoultella, Vibrio* and *Cronobacter*) results in MLD shown in Fig. 4(i). It is similar to MLD of $n = 9$ genera, but lacks the most recent HGT event (that contributed the longest matches). This indicate that this most recent HGT event did not manage to spread into the *Cronobacter* genus (at least to the 46 genomes we analyzed for this genus). The other two older HGT events are still visible on the MLD of all 10 genera. We find that these two HGT events contribute matches with different functional annotations, as shown in Fig. S11.

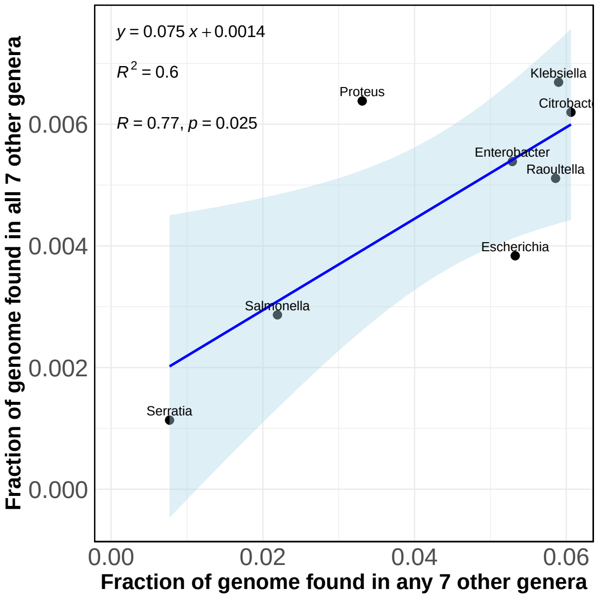

Figure S1: Average fraction of genome shared with all 7 other genera (at least one genome for each genus). *vs.* average fraction of genome shared with any 7 other genera (at least one genome in any genus).

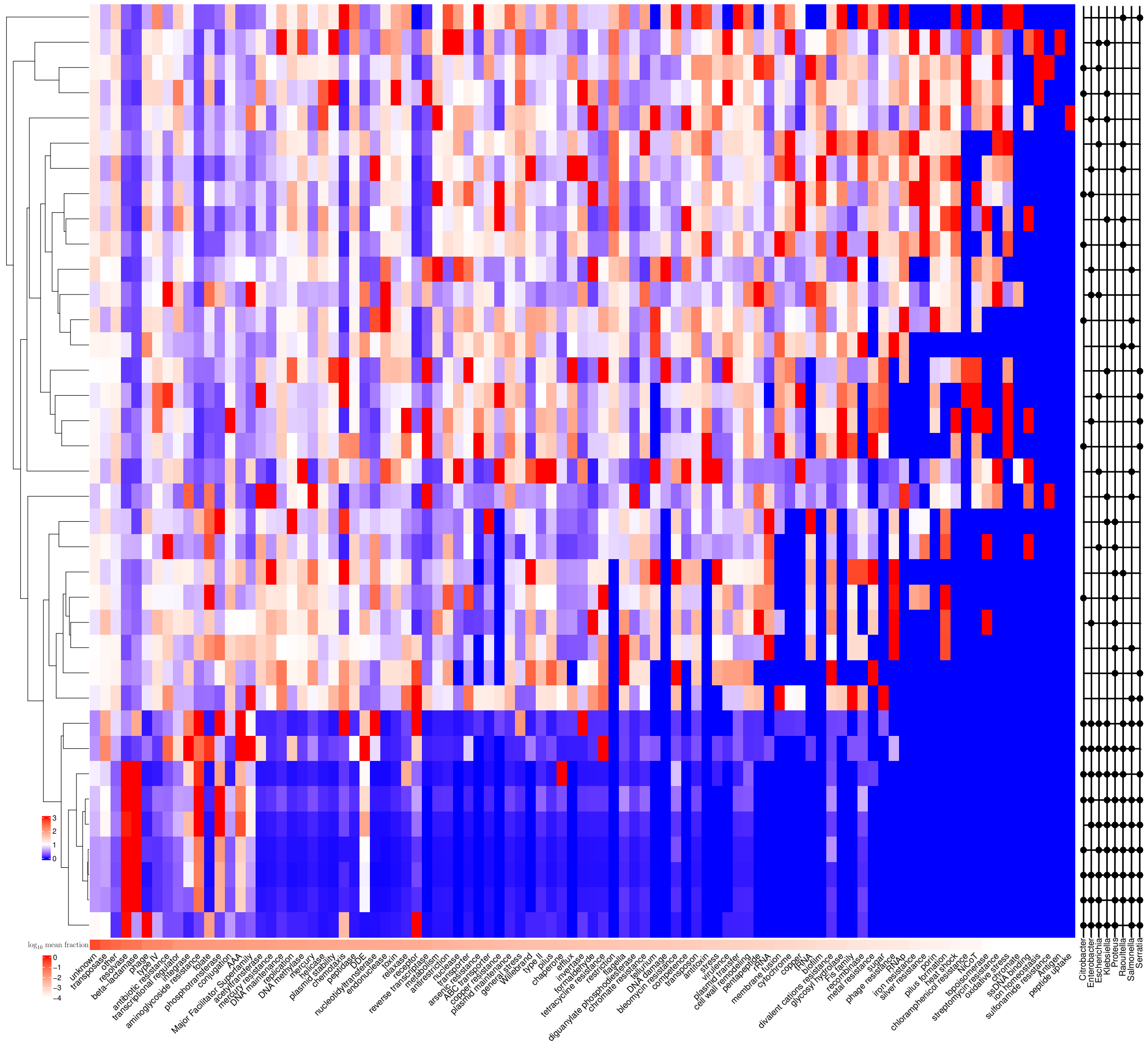

Figure S2: Fraction of annotations (columns) of genes located on the matches shared by sets of genera (rows). The fractions are normalized by the average fraction of all sets. To get the annotations, first for each set its match sequences were clustered using the `mmseqs easy-linclust -min-seq-id 0.5 -c 0.8 -cov-mode 1` command (Steinegger and Söding, 2017). Resulting representative sequences were annotated using `emapper.py -i -itype metagenome -m mmseqs` command (Cantalapiedra et al., 2021). The average annotation fractions are presented in the bottom with the shades of red on the logarithmic scale.

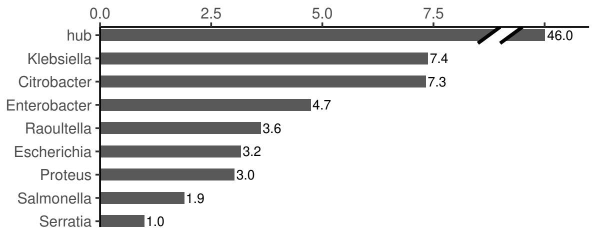

Figure S3: Calculated transferabilities of studied genera.

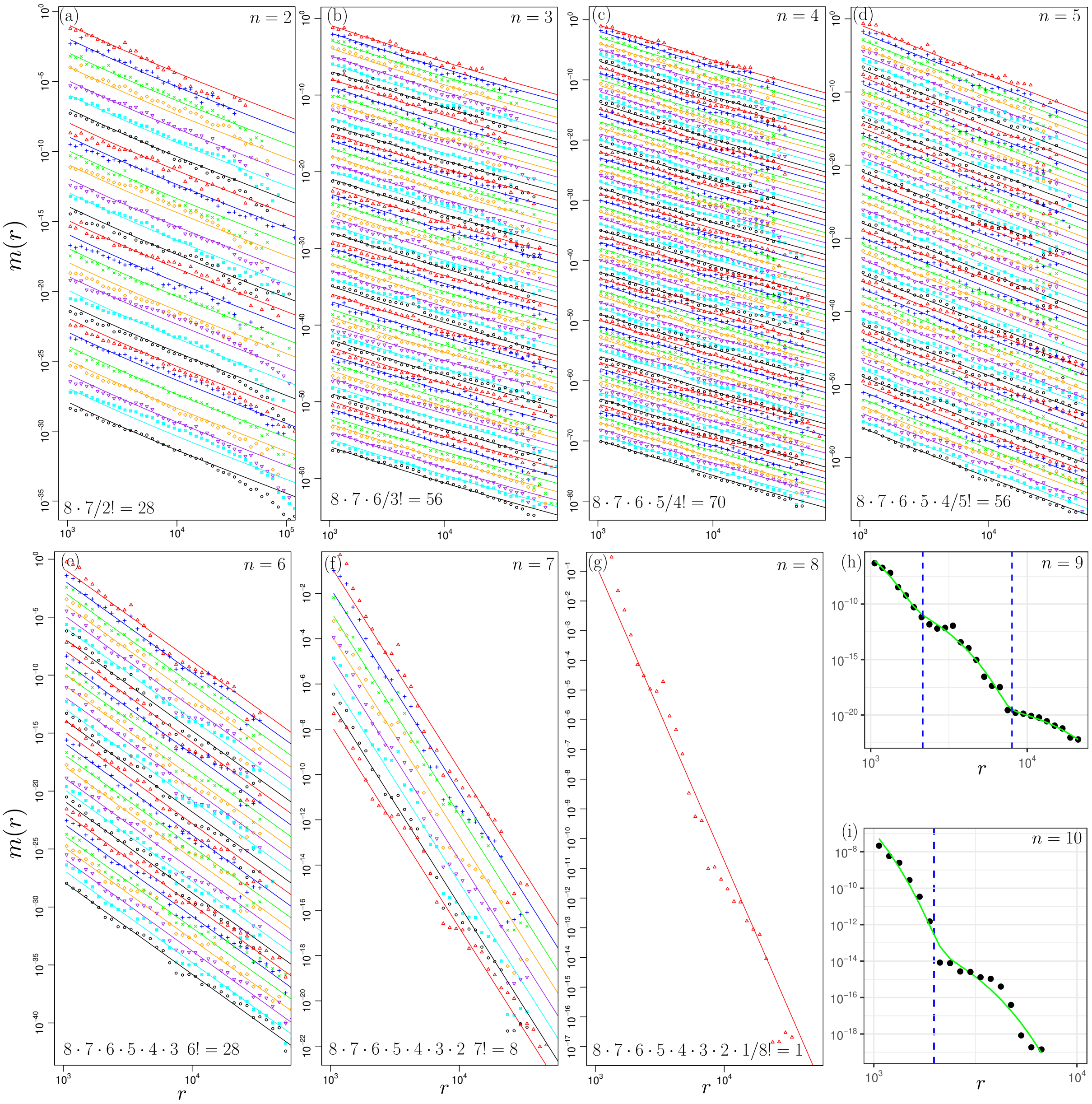

Figure S4: (a-g) MLDs of all comparisons of pairs ($n = 2$), trios ($n = 3$), quartets ($n = 4$) *etc* (see upper-right corners). Markers represent the empirical data and the lines represent the theoretical predictions. The MLDs are multiplies by constant prefactors for a better visibility, so the y-axes are arbitrary. For each set the empirical MLD and the theoretical one are multiplies by the same number. The number of considered sets for each n are shown in the lower-left corners. (h) MLD of $n = 9$ set: *Escherichia, Klebsiella, Salmonella, Enterobactervs. Citrobacter, Serratia, Proteus, Raoultella, Vibrio*. The green line is fit with 3 exponential functions and the dashed lines represent the crossover between the exponential functions. (i) MLD of $n = 10$ set: *Escherichia, Klebsiella, Salmonella, Enterobactervs. Citrobacter, Serratia, Proteus, Raoultella, Vibrio, Cronobacter*. The green line is fit with 2 exponential functions and the dashed line represents the crossover between the exponential functions.

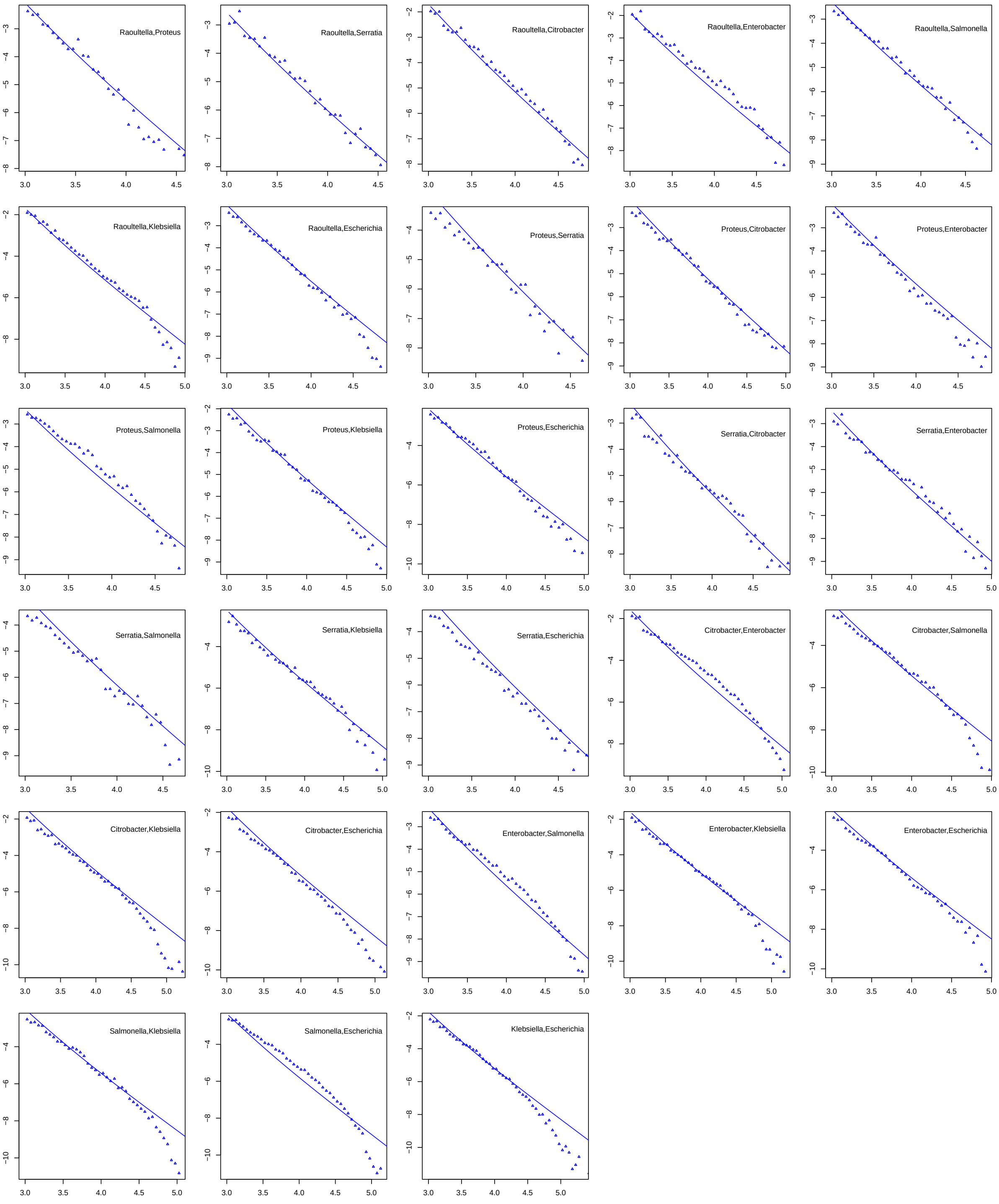

Figure S5: Here MLDs for $n = 2$ sets are presented on the log − log scale: $\log_{10} m(r)$ vs. $\log_{10} r$. The sets are indicated in the upper right corners. Points represent the empirical data, while the lines represent the prediction of the model.

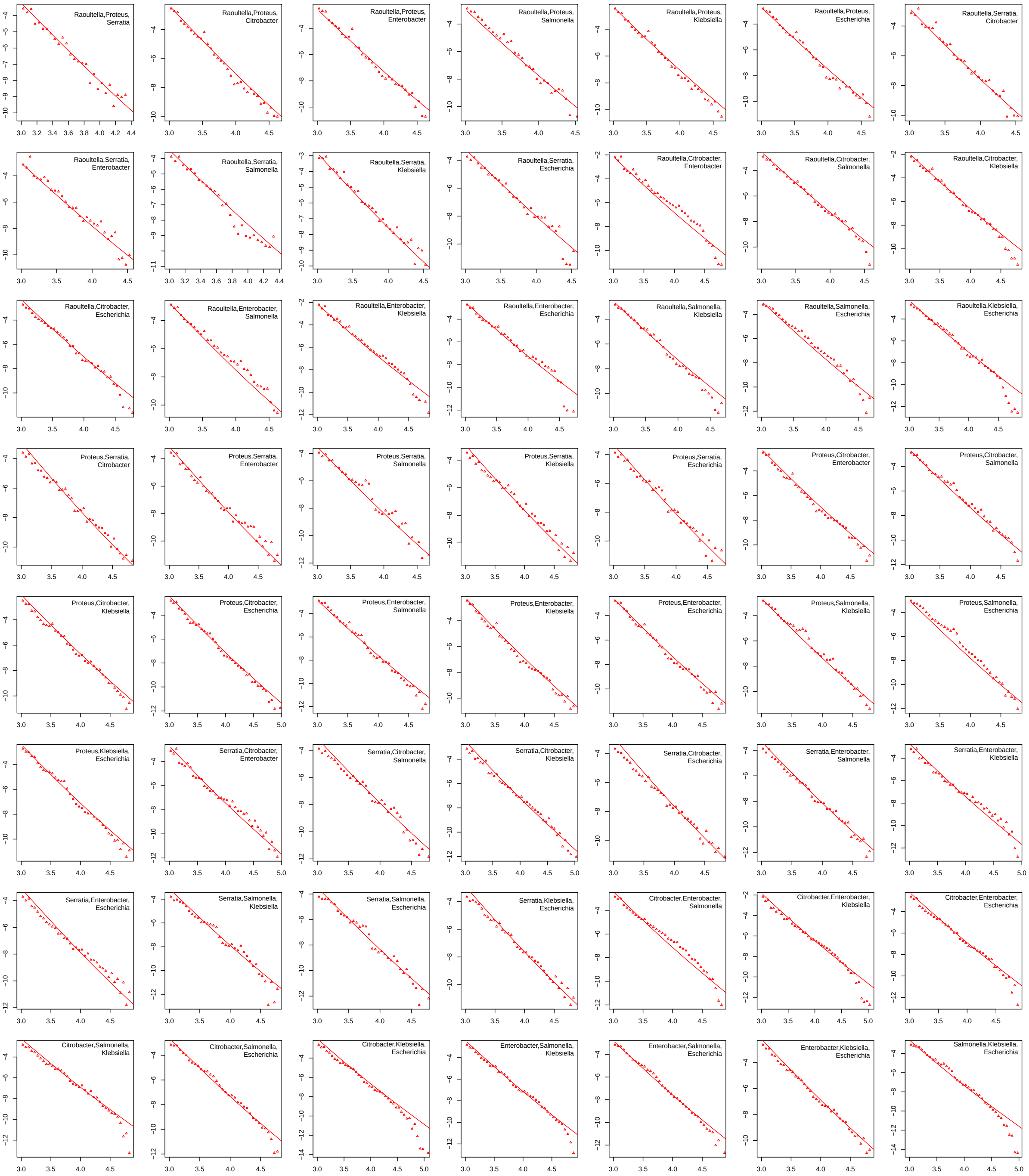

Figure S6: Here MLDs for $n = 3$ sets are presented on the log − log scale: $\log_{10} m(r)$ vs. $\log_{10} r$. The sets are indicated in the upper right corners. Points represent the empirical data, while the lines represent the prediction of the model.

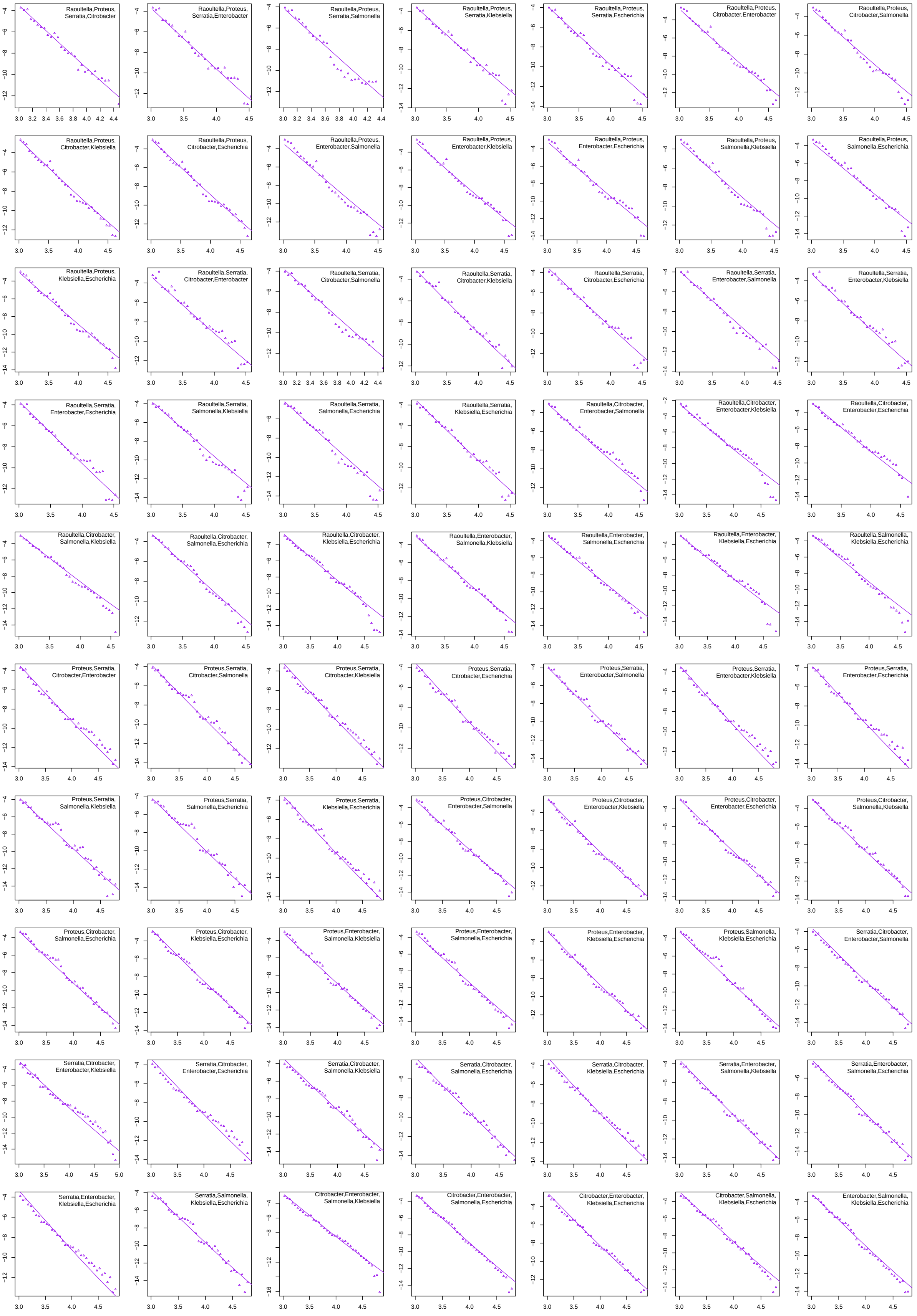

Figure S7: Here MLDs for $n = 4$ sets are presented on the $\log - \log$ scale: $\log_{10} m(r)$ vs. $\log_{10} r$. The sets are indicated in the upper right corners. Points represent the empirical data, while the lines represent the prediction of the model.

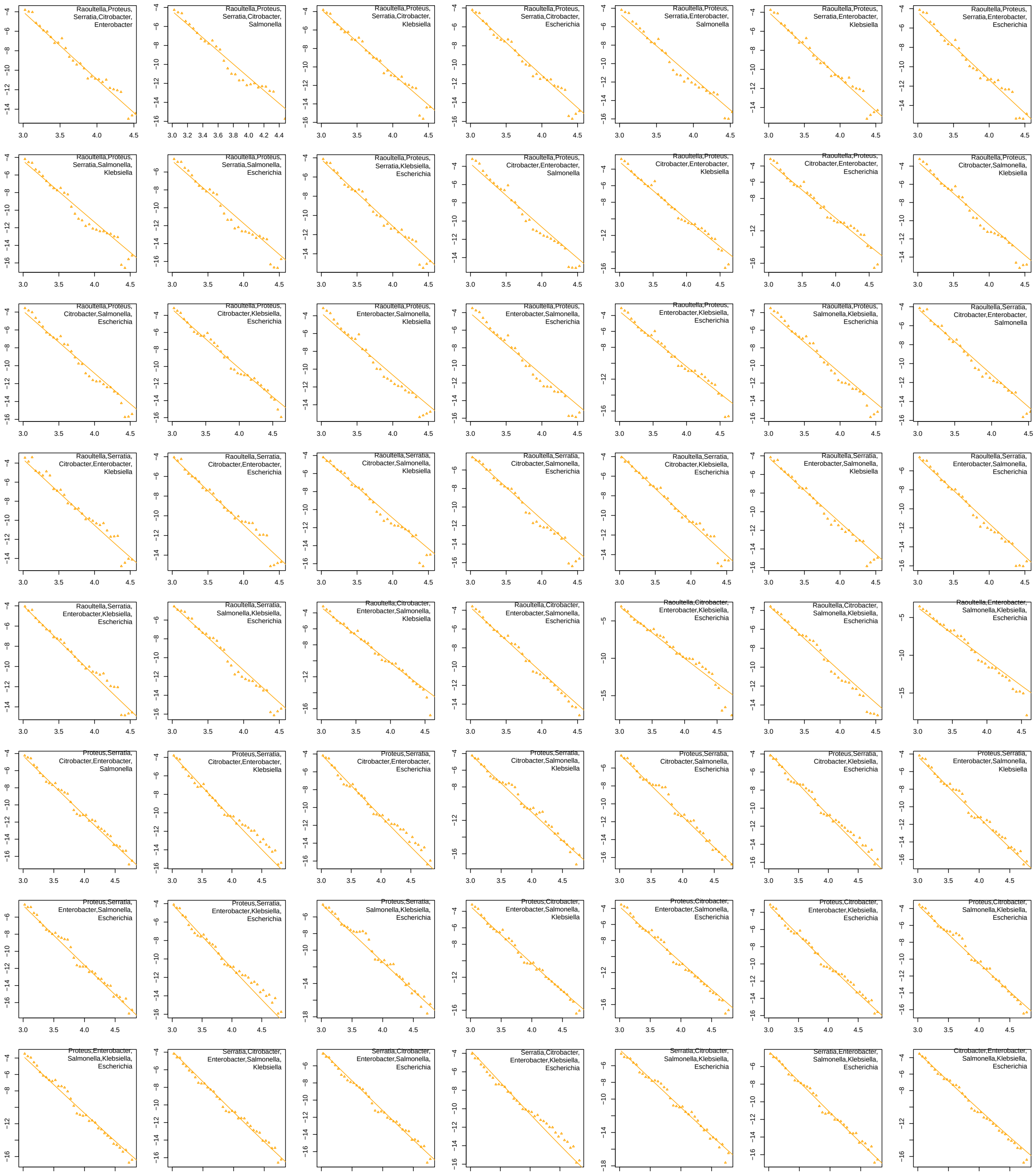

Figure S8: Here MLDs for $n = 5$ sets are presented on the $\log - \log$ scale: $\log_{10} m(r)$ vs. $\log_{10} r$. The sets are indicated in the upper right corners. Points represent the empirical data, while the lines represent the prediction of the model.

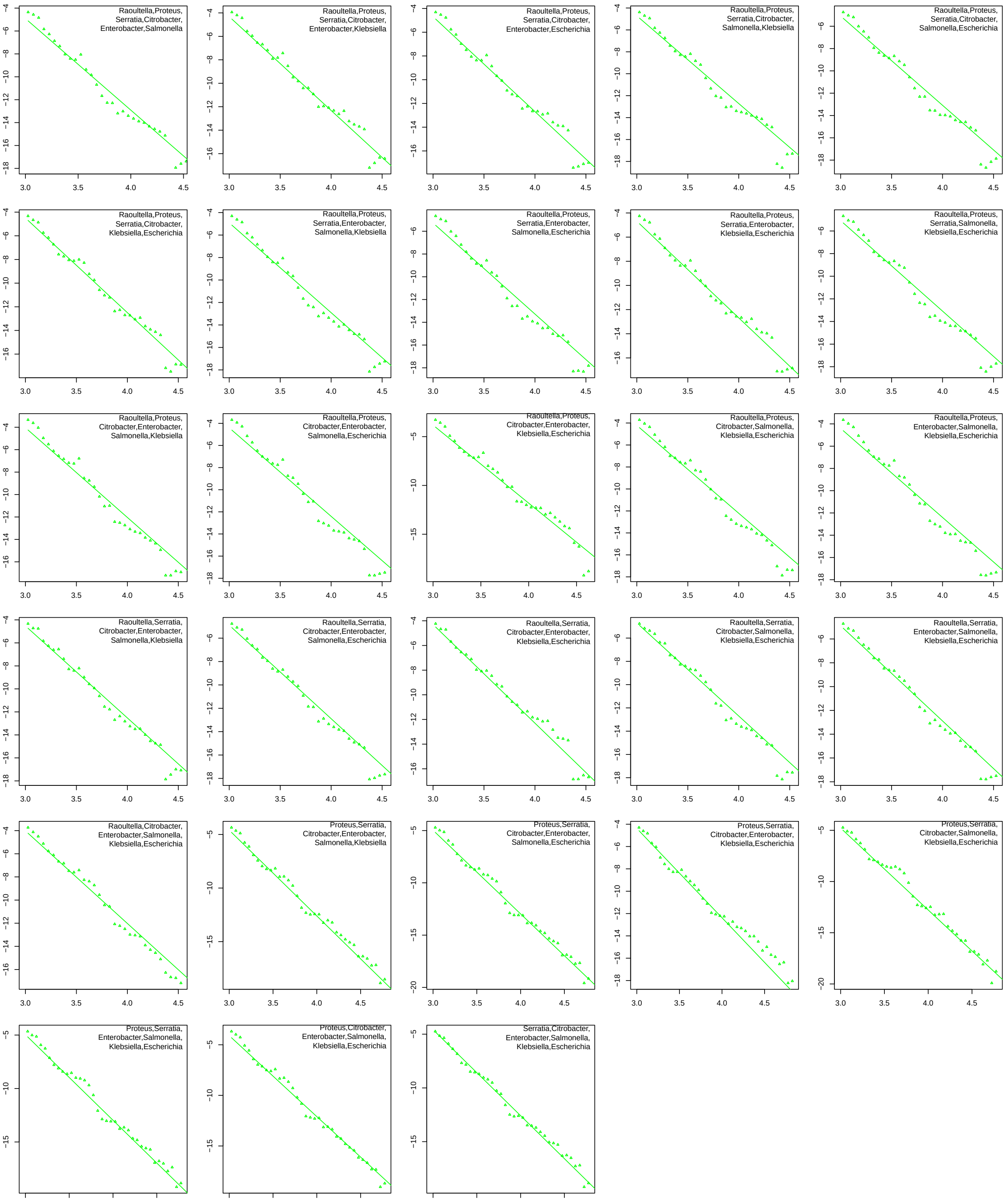

Figure S9: Here MLDs for $n = 6$ sets are presented on the log − log scale: $\log_{10} m(r)$ vs. $\log_{10} r$. The sets are indicated in the upper right corners. Points represent the empirical data, while the lines represent the prediction of the model.

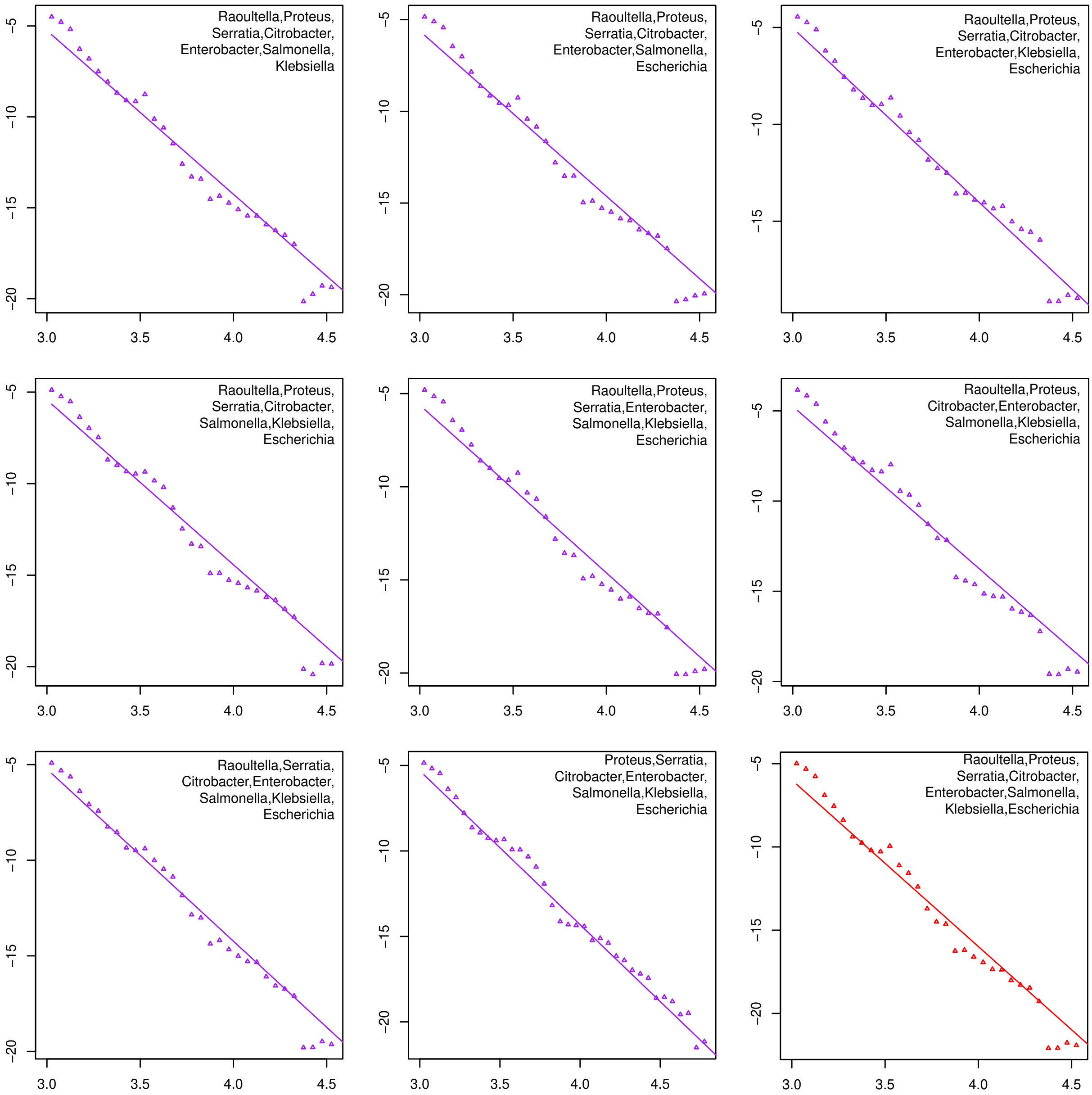

Figure S10: Here MLDs for $n = 7$ and $n = 8$ (last plot) sets are presented on the $\log - \log$ scale: $\log_{10} m(r)$ vs. $\log_{10} r$. The sets are indicated in the upper right corners. Points represent the empirical data, while the lines represent the prediction of the model.

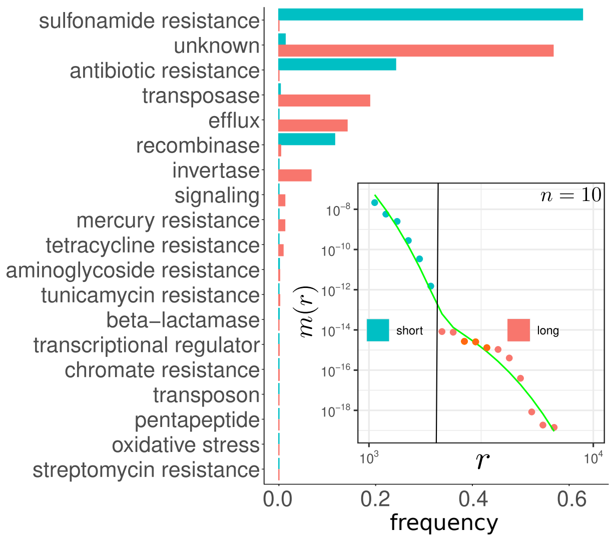

Figure S11: Functional analysis of matches shared by 10 genera: *Escherichia, Klebsiella, Salmonella, Enterobacter, Citrobacter, Serratia, Proteus, Raoultella, Vibrio* and *Cronobacter*. The MLD of the matches can be fitted with two exponential functions (see inset and Fig. 4(i)). Short matches (green dots and bars) and long matches (red dots and bars) have different functional annotations.

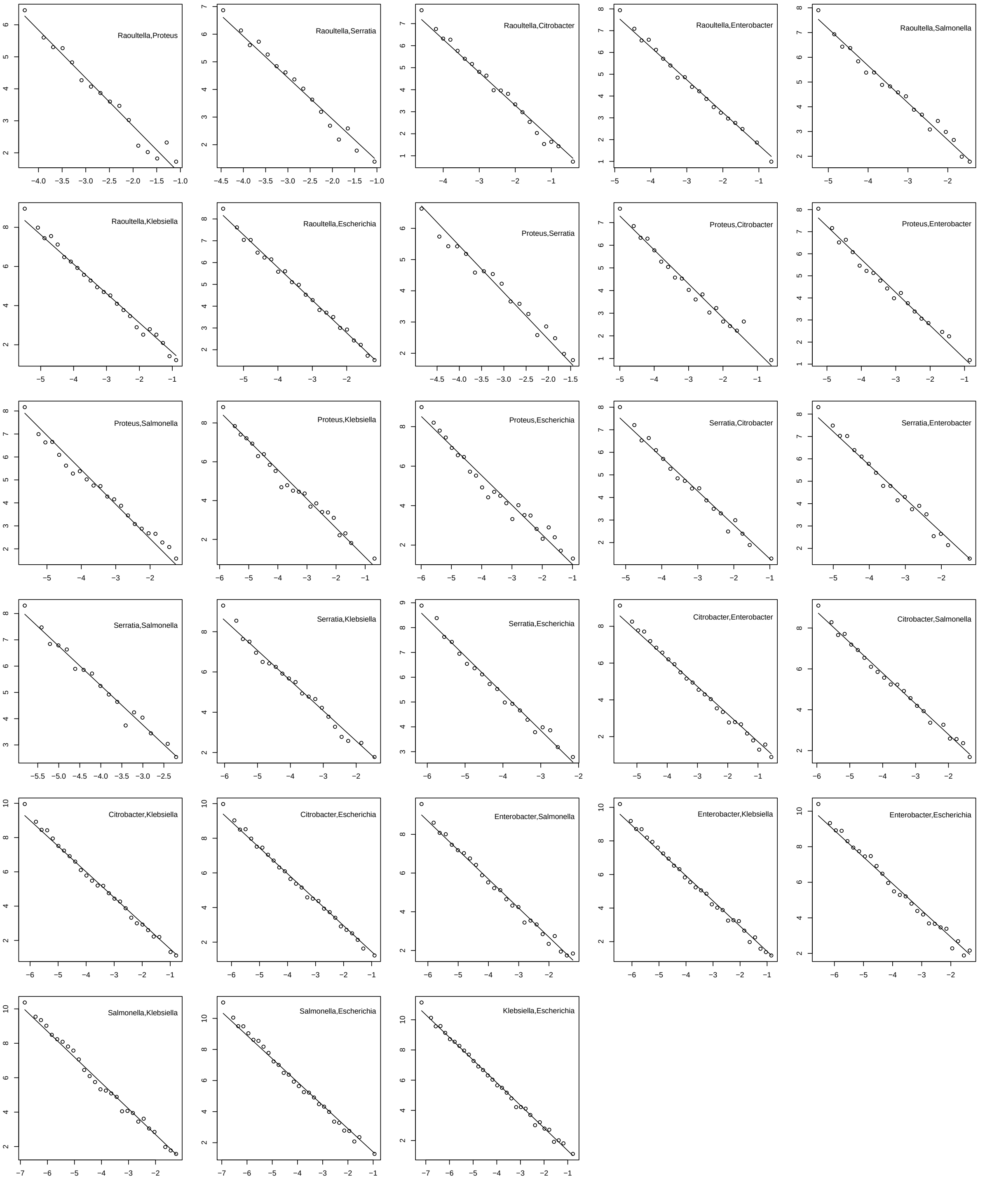

Figure S12: Here distributions of sequences abundances fractions are presented for $n = 2$. After clustering the matches we calculate the sizes of the clusters and divide by the total number of comparisons for each set (product of number of genomes for each genus in the set) to get the abundance fraction of each sequence—the probability to obtain this sequence taking $n = |s| = 2$ random genomes from the set s, each genome from a different genus. The probability density of this abundance fraction is presented on the plots for all pairs of genera (annotated in the upper right corners) on the $\log_{10} - \log_{10}$ scale. Points represent the empirical data, while the lines represent the power-law with $-3/2$ exponent (we don't have a theory that predicts this exponent).

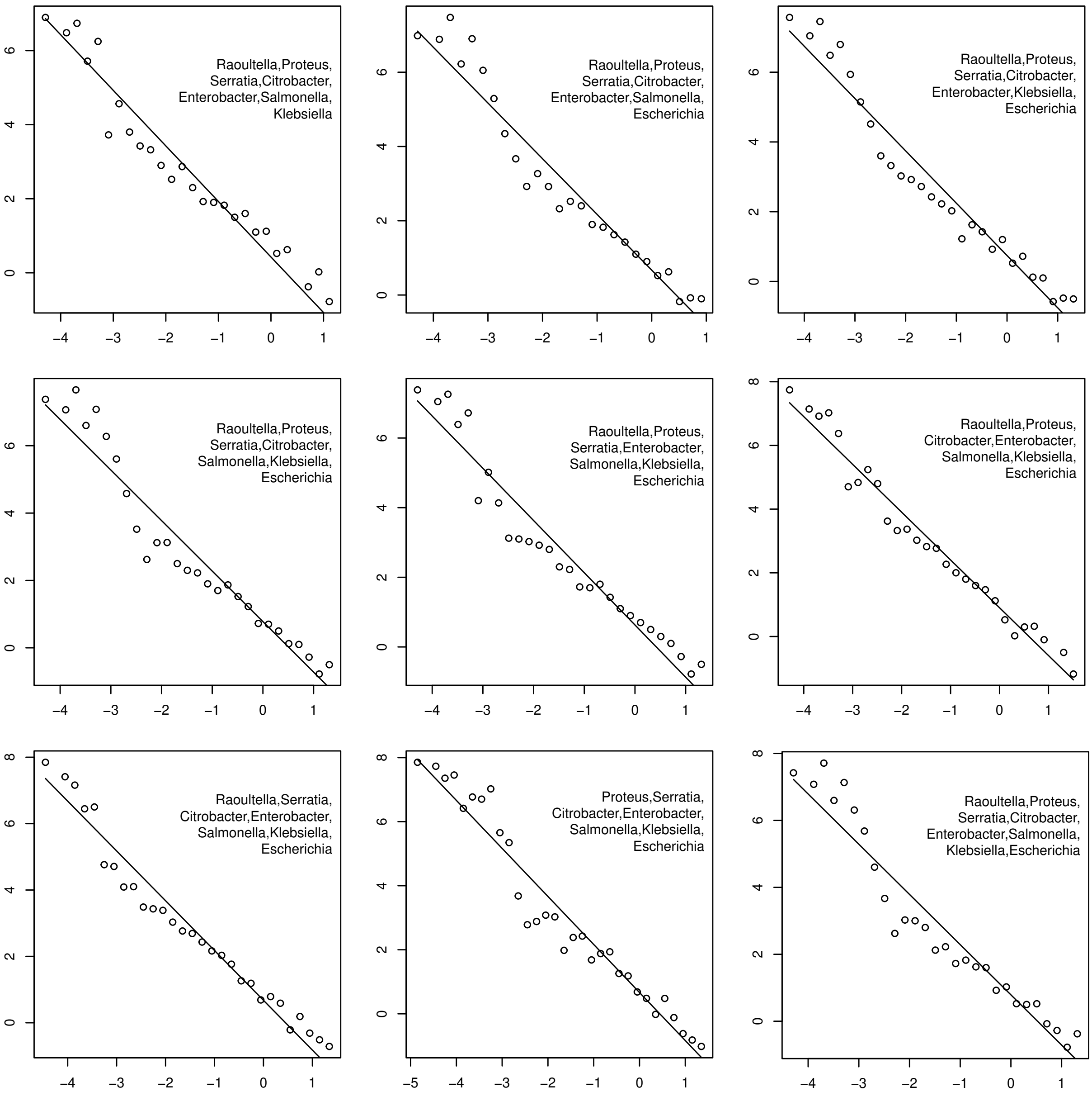

Figure S13: The same as in Fig. S12, but for sets of genera with $n = |s| = 7$ and 8 (last plot).

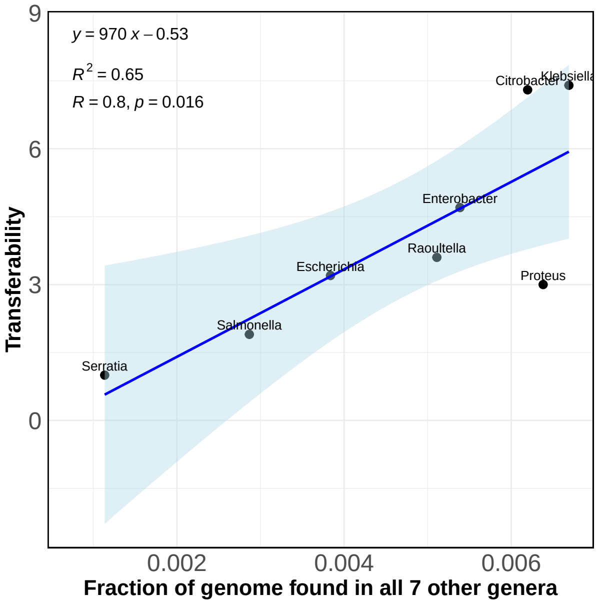

Figure S14: Calculated transferabilities of the eight studied genera *vs.* average fraction of genome shared with 7 other genera (at least one genome for each genus).

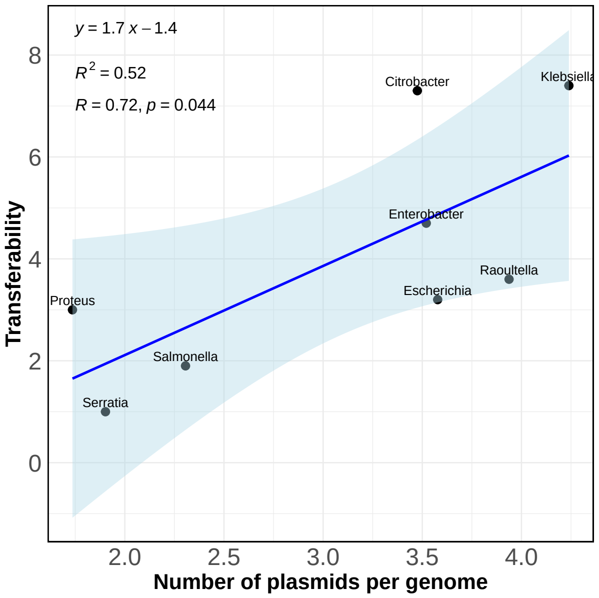

Figure S15: Calculated transferabilities of the eight studied genera *vs.* average number of plasmids per genome.

References

Arndt, P. F. (2019), 'Sequential and continuous time stick-breaking', *Journal of Statistical Mechanics: Theory and Experiment* **2019**(6), 064003.

Bapteste, E., O'Malley, M. A., Beiko, R. G., Ereshefsky, M., Gogarten, J. P. et al. (2009), 'Prokaryotic evolution and the tree of life are two different things', *Biology direct* **4**(1), 34.

Battistuzzi, F. U., Feijao, A. and Hedges, S. B. (2004), 'A genomic timescale of prokaryote evolution: insights into the origin of methanogenesis, phototrophy, and the colonization of land', *BMC evolutionary biology* **4**(1), 1–14.

Benoit, G., Raguideau, S., James, R., Phillippy, A. M., Chikhi, R. et al. (2024), 'High-quality metagenome assembly from long accurate reads with metamdbg', *Nature Biotechnology* **42**(9), 1378–1383.

Cantalapiedra, C. P., Hernández-Plaza, A., Letunic, I., Bork, P. and Huerta-Cepas, J. (2021), 'eggnog-mapper v2: functional annotation, orthology assignments, and domain prediction at the metagenomic scale', *Molecular biology and evolution* **38**(12), 5825–5829.

Carr, V. R., Shkoporov, A., Hill, C., Mullany, P. and Moyes, D. L. (2021), 'Probing the mobilome: discoveries in the dynamic microbiome', *Trends in Microbiology* **29**(2), 158–170.

Choi, S. C., Rasmussen, M. D., Hubisz, M. J., Gronau, I., Stanhope, M. J. et al. (2012), 'Replacing and additive horizontal gene transfer in streptococcus', *Molecular biology and evolution* **29**(11), 3309–3320.

Colombi, E., Perry, B. J., Sullivan, J. T., Bekuma, A. A., Terpolilli, J. J. et al. (2021), 'Comparative analysis of integrative and conjugative mobile genetic elements in the genus mesorhizobium', *Microbial Genomics* **7**(10), 000657.

Cordero, O. X. and Polz, M. F. (2014), 'Explaining microbial genomic diversity in light of evolutionary ecology', *Nature Reviews Microbiology* **12**(4), 263–273.

Corel, E., Lopez, P., Méheust, R. and Bapteste, E. (2016), 'Network-thinking: graphs to analyze microbial complexity and evolution', *Trends in Microbiology* **24**(3), 224–237.

Costa, S. S., Guimarães, L. C., Silva, A., Soares, S. C. and Baraúna, R. A. (2020), 'First steps in the analysis of prokaryotic pan-genomes', *Bioinformatics and biology insights* **14**, 1177932220938064.

Cury, J., Touchon, M. and Rocha, E. P. (2017), 'Integrative and conjugative elements and their hosts: composition, distribution and organization', *Nucleic acids research* **45**(15), 8943–8956.

Dagan, T., Artzy-Randrup, Y. and Martin, W. (2008), 'Modular networks and cumulative impact of lateral transfer in prokaryote genome evolution', *Proceedings of the National Academy of Sciences* **105**(29), 10039–10044.

Darwin, C. (1859), *The Origin of Species by Means of Natural Selection, Or, The Preservation of Favoured Races in the Struggle for Life*, Books, Incorporated, Pub.

Didelot, X., Walker, A. S., Peto, T. E., Crook, D. W. and Wilson, D. J. (2016), 'Within-host evolution of bacterial pathogens', *Nature Reviews Microbiology* **14**(3), 150–162.

Dixit, P. D., Pang, T. Y., Studier, F. W. and Maslov, S. (2015), 'Recombinant transfer in the basic genome of escherichia coli', *Proceedings of the National Academy of Sciences* **112**(29), 9070–9075.

Dmitrijeva, M., Tackmann, J., Matias Rodrigues, J. F., Huerta-Cepas, J., Coelho, L. P. et al. (2024), 'A global survey of prokaryotic genomes reveals the eco-evolutionary pressures driving horizontal gene transfer', *Nature Ecology & Evolution* **8**(5), 986–998.

Doolittle, W. F. (1999), 'Phylogenetic classification and the universal tree', *Science* **284**(5423), 2124–2128.

Drake, J. W. (1991), 'A constant rate of spontaneous mutation in DNA-based microbes.', *Proceedings of the National Academy of Sciences* **88**(16), 7160–7164.

Duchêne, S., Holt, K. E., Weill, F.-X., Le Hello, S., Hawkey, J. et al. (2016), 'Genome-scale rates of evolutionary change in bacteria', *Microbial genomics* **2**(11).

Evans, D. R., Griffith, M. P., Sundermann, A. J., Shutt, K. A., Saul, M. I. et al. (2020), 'Systematic detection of horizontal gene transfer across genera among multidrug-resistant bacteria in a single hospital', *eLife* **9**, e53886.

Gerber, F. (2021), 'optimparallel: An r package providing a parallel version of the l-bfgs-b optimization method', *The R Journal* **13**(2), 424.

Groussin, M., Poyet, M., Sistiaga, A., Kearney, S. M., Moniz, K. et al. (2021), 'Elevated rates of horizontal gene transfer in the industrialized human microbiome', *Cell* **184**(8), 2053–2067.

Harris, K. and Nielsen, R. (2013), 'Inferring demographic history from a spectrum of shared haplotype lengths', *PLoS genetics* **9**(6), e1003521.

Johnson, C. M. and Grossman, A. D. (2015), 'Integrative and conjugative elements (ICEs): what they do and how they work', *Annual review of genetics* **49**(1), 577–601.

Khaledian, E., Brayton, K. A. and Broschat, S. L. (2020), 'A systematic approach to bacterial phylogeny using order level sampling and identification of HGT using network science', *Microorganisms* **8**(2), 312.

Kloub, L., Gosselin, S., Graf, J., Gogarten, J. P. and Bansal, M. S. (2024), 'Investigating additive and replacing horizontal gene transfers using phylogenies and whole genomes', *Genome Biology and Evolution* **16**(9), evae180.

Kumar, S., Stecher, G., Suleski, M. and Hedges, S. B. (2017), 'Timetree: a resource for timelines, timetrees, and divergence times', *Molecular biology and evolution* **34**(7), 1812–1819.

Kunin, V., Goldovsky, L., Darzentas, N. and Ouzounis, C. A. (2005), 'The net of life: Reconstructing the microbial phylogenetic network', *Genome Research* **15**(7), 954–959.

Li, C., Chen, J. and Li, S. C. (2020), 'Understanding horizontal gene transfer network in human gut microbiota', *Gut Pathogens* **12**(1), 33.

Liu, Y., Zhang, L. Y. and Li, J. (2019), 'Fast detection of maximal exact matches via fixed sampling of query k-mers and bloom filtering of index k-mers', *Bioinformatics* **35**(22), 4560–4567.

López Sánchez, A. and Lafond, M. (2024), 'Predicting horizontal gene transfers with perfect transfer networks', *Algorithms for Molecular Biology* **19**(1), 6.

Massip, F. and Arndt, P. F. (2013), 'Neutral evolution of duplicated DNA: an evolutionary stick-breaking process causes scale-invariant behavior', *Physical review letters* **110**(14), 148101.

Massip, F., Sheinman, M., Schbath, S. and Arndt, P. F. (2015), 'How evolution of genomes is reflected in exact DNA sequence match statistics', *Molecular biology and evolution* **32**(2), 524–535.

Massip, F., Sheinman, M., Schbath, S. and Arndt, P. F. (2016), 'Comparing the statistical fate of paralogous and orthologous sequences', *Genetics* **204**(2), 475–482.

Novichkov, P. S., Omelchenko, M. V., Gelfand, M. S., Mironov, A. A., Wolf, Y. I. et al. (2004), 'Genome-wide molecular clock and horizontal gene transfer in bacterial evolution', *Journal of bacteriology* **186**(19), 6575–6585.

Ochman, H., Elwyn, S. and Moran, N. A. (1999), 'Calibrating bacterial evolution', *Proceedings of the National Academy of Sciences* **96**(22), 12638–12643.

Ochman, H., Lawrence, J. G. and Groisman, E. A. (2000), 'Lateral gene transfer and the nature of bacterial innovation', *nature* **405**(6784), 299–304.

O'Leary, N. A., Wright, M. W., Brister, J. R., Ciufo, S., Haddad, D. et al. (2016), 'Reference sequence (RefSeq) database at NCBI: current status, taxonomic expansion, and functional annotation', *Nucleic acids research* **44**(D1), D733–D745.

Partridge, S. R., Kwong, S. M., Firth, N. and Jensen, S. O. (2018), 'Mobile genetic elements associated with antimicrobial resistance', *Clinical microbiology reviews* **31**(4), 10–1128.

Quandt, R. E. (1964), 'Old and mew methods op estimation and the pareto distribution', *Metrika* **10**.

Ravenhall, M., Škunca, N., Lassalle, F. and Dessimoz, C. (2015), 'Inferring horizontal gene transfer', *PLoS computational biology* **11**(5), e1004095.

Redondo-Salvo, S., Fernández-López, R., Ruiz, R., Vielva, L., de Toro, M. et al. (2020), 'Pathways for horizontal gene transfer in bacteria revealed by a global map of their plasmids', *Nature communications* **11**(1), 3602.

Sakoparnig, T., Field, C. and van Nimwegen, E. (2021), 'Whole genome phylogenies reflect the distributions of recombination rates for many bacterial species', *eLife* **10**, e65366.

Sayers, E. W., Bolton, E. E., Brister, J. R., Canese, K., Chan, J. et al. (2023), 'Database resources of the national center for biotechnology information in 2023', *Nucleic acids research* **51**(D1), D29–D38.

Scrucca, L. (2013), 'Ga: A package for genetic algorithms in r', *Journal of Statistical Software* **53**, 1–37.

Shapiro, J. T., Zorea, A., Kav, A. B., Ontiveros, V. J., Mizrahi, I. et al. (2023), 'Multilayer networks of plasmid genetic similarity reveal potential pathways of gene transmission', *The ISME Journal* **17**(5), 649–659.

Sheinman, M., Arkhipova, K., Arndt, P. F., Dutilh, B. E., Hermsen, R. et al. (2021), 'Identical sequences found in distant genomes reveal frequent horizontal transfer across the bacterial domain', *eLife* **10**, e62719.

Sheinman, M., Arndt, P. F. and Massip, F. (2024), 'Modeling the mosaic structure of bacterial genomes to infer their evolutionary history', *Proceedings of the National Academy of Sciences* **121**(13), e2313367121.

Sheinman, M., Stentella, T., Etheimer, P., Massip, F. and Arndt, P. F. (2025), https://github.com/mishashe/networkHGT.

Siefert, J. L. (2009), 'Defining the mobilome', *Horizontal gene transfer: Genomes in flux* pp. 13–27.

Smillie, C. S., Smith, M. B., Friedman, J., Cordero, O. X., David, L. A. et al. (2011), 'Ecology drives a global network of gene exchange connecting the human microbiome', *Nature* **480**(7376), 241–244.

Šorfová, P., Škeříková, A. and Hypša, V. (2008), 'An effect of 16s rRNA intercistronic variability on coevolutionary analysis in symbiotic bacteria: molecular phylogeny of arsenophonus triatominarum', *Systematic and applied microbiology* **31**(2), 88–100.

Soucy, S. M., Huang, J. and Gogarten, J. P. (2015), 'Horizontal gene transfer: building the web of life', *Nature Reviews Genetics* **16**(8), 472–482.

Steinegger, M. and Söding, J. (2017), 'Mmseqs2 enables sensitive protein sequence searching for the analysis of massive data sets', *Nature biotechnology* **35**(11), 1026–1028.

Tettelin, H., Riley, D., Cattuto, C. and Medini, D. (2008), 'Comparative genomics: the bacterial pangenome', *Current opinion in microbiology* **11**(5), 472–477.

Treangen, T. J. and Rocha, E. P. (2011), 'Horizontal transfer, not duplication, drives the expansion of protein families in prokaryotes', *PLoS genetics* **7**(1), e1001284.

Zatyka, M. and Thomas, C. M. (1998), 'Control of genes for conjugative transfer of plasmids and other mobile elements', *FEMS microbiology reviews* **21**(4), 291–319.

Zhao, S., Lieberman, T. D., Poyet, M., Kauffman, K. M., Gibbons, S. M. et al. (2019), 'Adaptive evolution within gut microbiomes of healthy people', *Cell host & microbe* **25**(5), 656–667.

Zheng, Q., Li, L., Yin, X., Che, Y. and Zhang, T. (2023), 'Is ICE hot? a genomic comparative study reveals integrative and conjugative elements as "hot" vectors for the dissemination of antibiotic resistance genes', *Msystems* **8**(6), e00178–23.

Zhu, C., Byrd, R. H., Lu, P. and Nocedal, J. (1997), 'Algorithm 778: L-bfgs-b: Fortran subroutines for large-scale bound-constrained optimization', *ACM Transactions on mathematical software (TOMS)* **23**(4), 550–560.

Ziff, R. M. and McGrady, E. (1985), 'The kinetics of cluster fragmentation and depolymerisation', *Journal of Physics A: Mathematical and General* **18**(15), 3027.

Zuckerkandl, E. and Pauling, L. (1965), Evolutionary divergence and convergence in proteins, *in* 'Evolving genes and proteins', Elsevier, pp. 97–166.
