# Supplementary material for: Reconstructing the Network of Horizontal Gene Exchange in Bacteria to Differentiate Direct and Indirect Transfers
Source: Genome Biol Evol. 2026 Apr 18;18(5):evag099. doi: 10.1093/gbe/evag099 (PMC13148533; doi:10.1093/gbe/evag099)
Supplement: evag099_Supplementary_Data [file evag099_supplementary_data.zip › supplementary_figures.pdf]

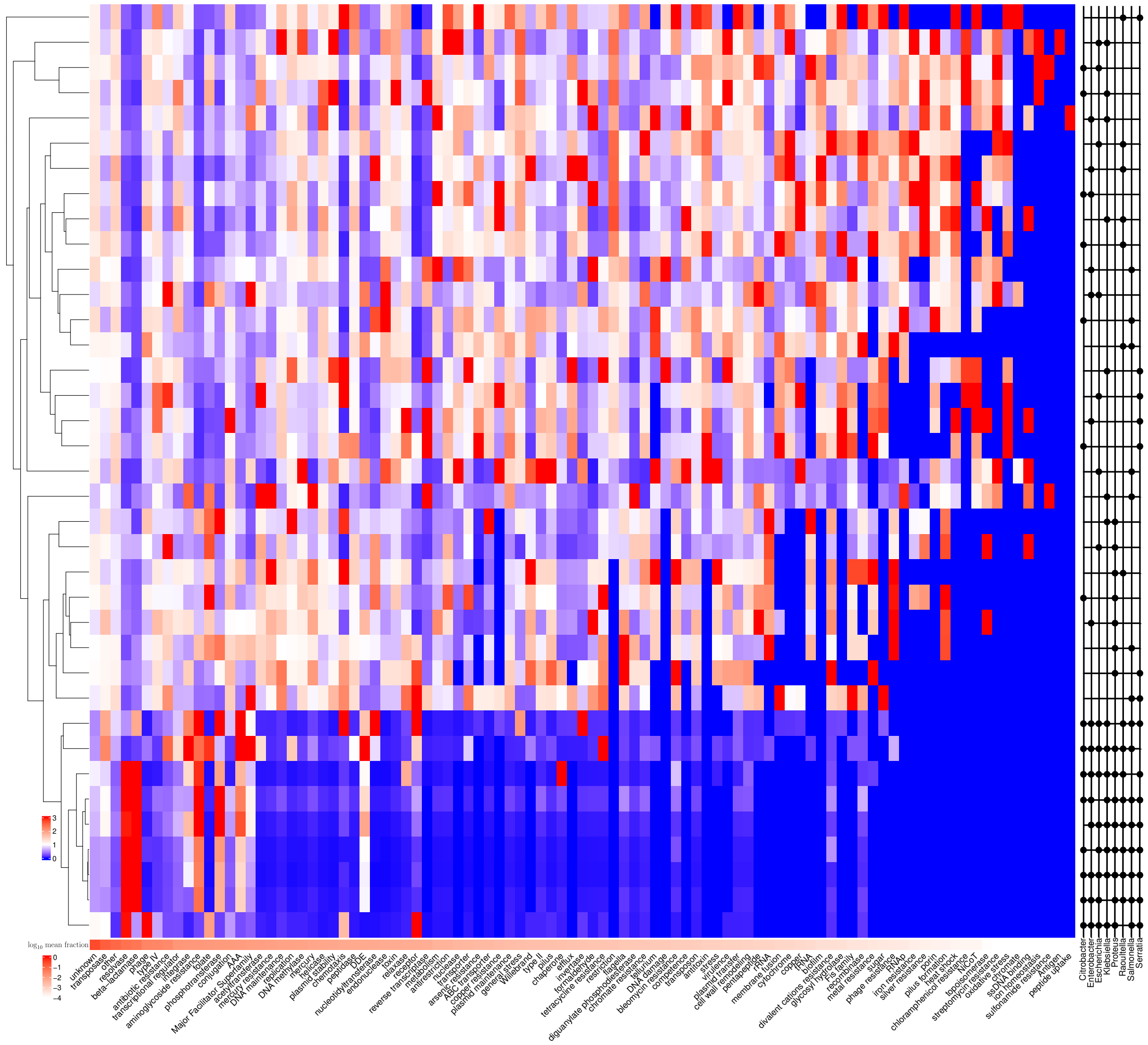

Figure S2: Fraction of annotations (columns) of genes located on the matches shared by sets of genera (rows). The fractions are normalized by the average fraction of all sets. To get the annotations, first for each set its match sequences were clustered using the `mmseqs easy-linclust -min-seq-id 0.5 -c 0.8 -cov-mode 1` command (Steinegger and Söding, 2017). Resulting representative sequences were annotated using `emapper.py -i -itype metagenome -m mmseqs` command (Cantalapiedra et al., 2021). The average annotation fractions are presented in the bottom with the shades of red on the logarithmic scale.

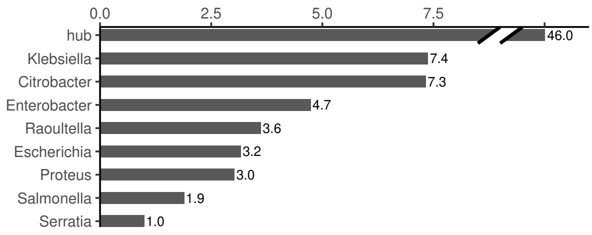

Figure S3: Calculated transferabilities of studied genera.

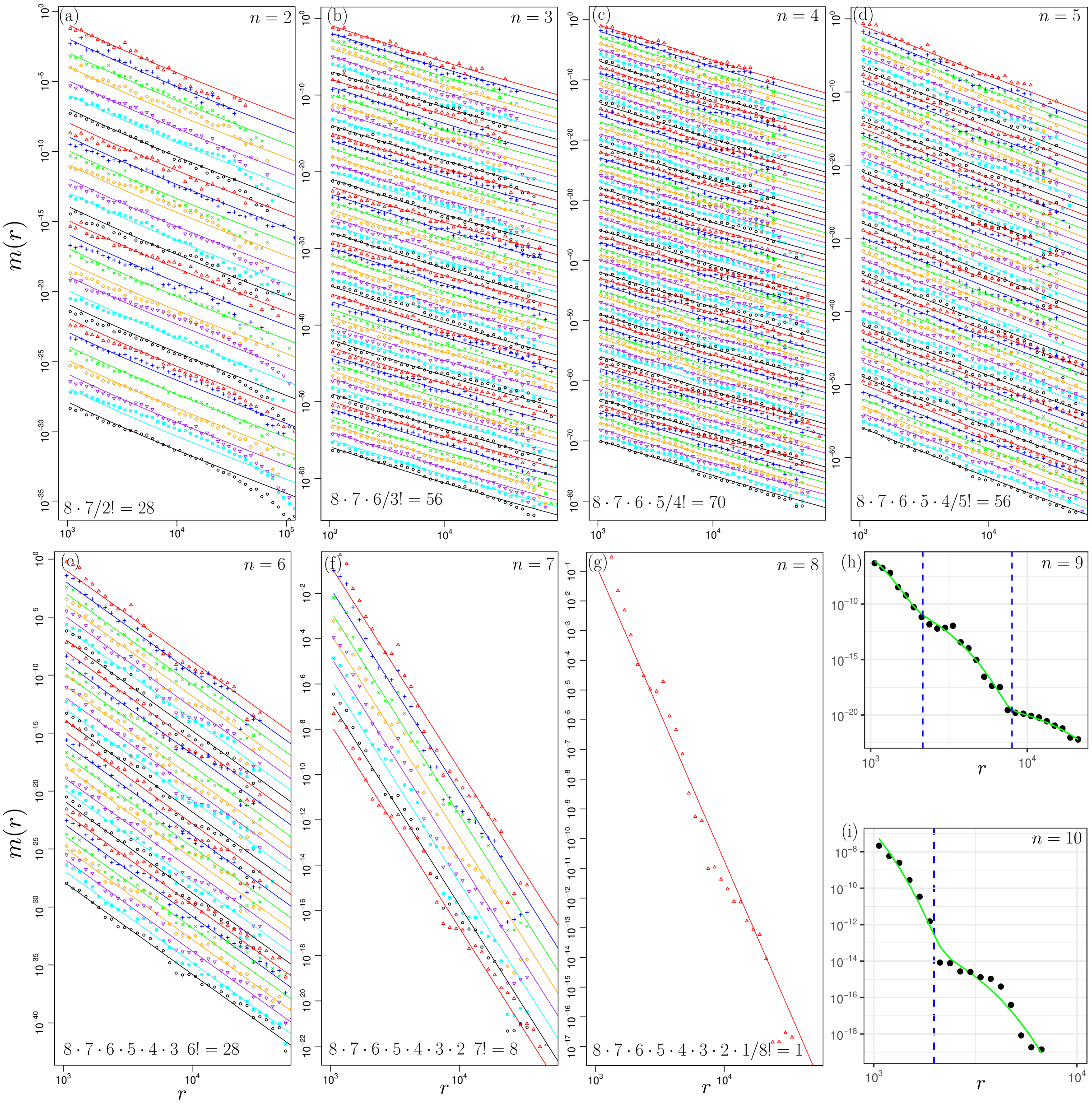

Figure S4: (a-g) MLDs of all comparisons of pairs ($n = 2$), trios ($n = 3$), quartets ($n = 4$) *etc* (see upper-right corners). Markers represent the empirical data and the lines represent the theoretical predictions. The MLDs are multiplies by constant prefactors for a better visibility, so the y-axes are arbitrary. For each set the empirical MLD and the theoretical one are multiplies by the same number. The number of considered sets for each n are shown in the lower-left corners. (h) MLD of $n = 9$ set: *Escherichia, Klebsiella, Salmonella, Enterobactervs. Citrobacter, Serratia, Proteus, Raoultella, Vibrio*. The green line is fit with 3 exponential functions and the dashed lines represent the crossover between the exponential functions. (i) MLD of $n = 10$ set: *Escherichia, Klebsiella, Salmonella, Enterobactervs. Citrobacter, Serratia, Proteus, Raoultella, Vibrio, Cronobacter*. The green line is fit with 2 exponential functions and the dashed line represents the crossover between the exponential functions.

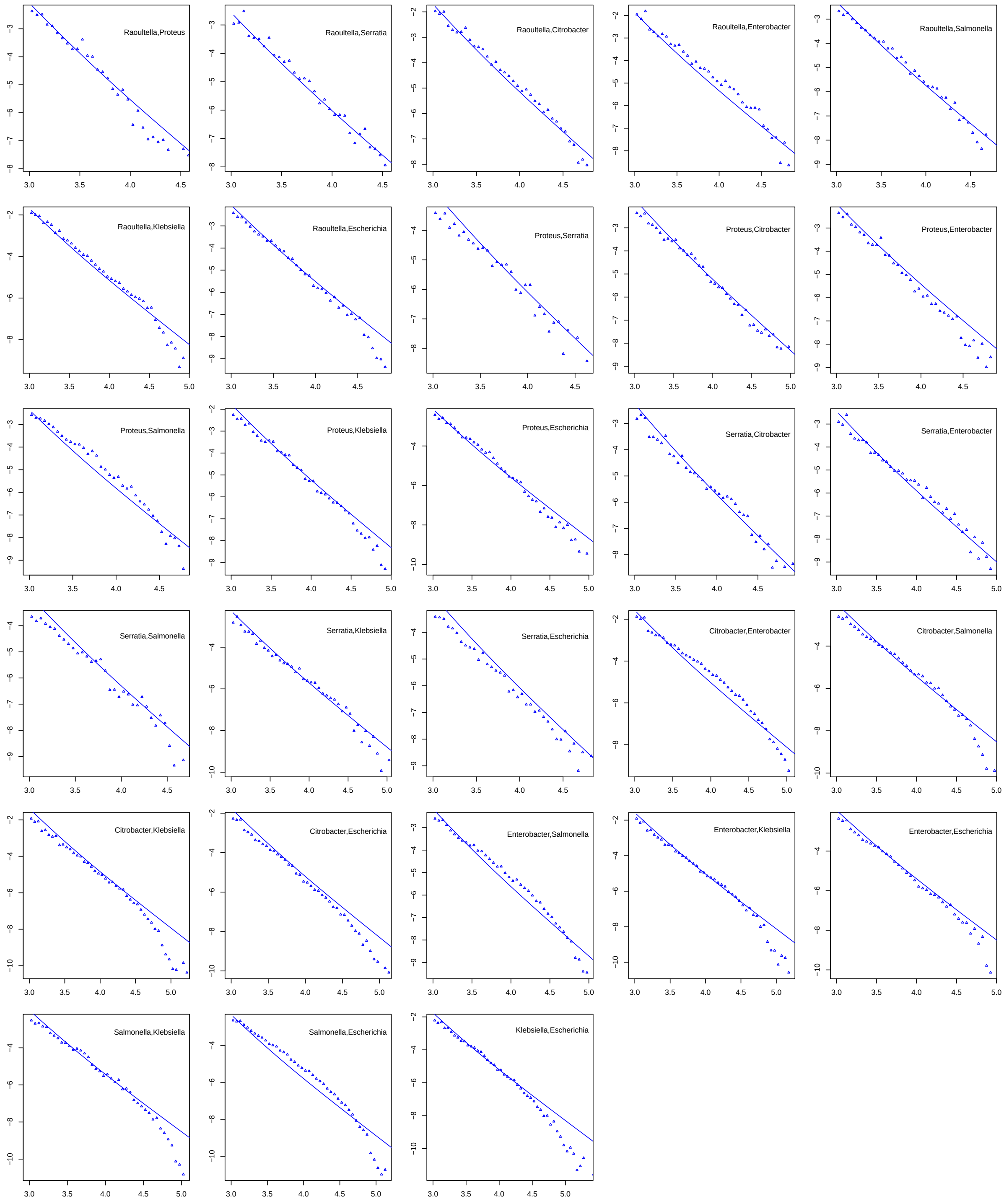

Figure S5: Here MLDs for $n = 2$ sets are presented on the log − log scale: $\log_{10} m(r)$ vs. $\log_{10} r$. The sets are indicated in the upper right corners. Points represent the empirical data, while the lines represent the prediction of the model.

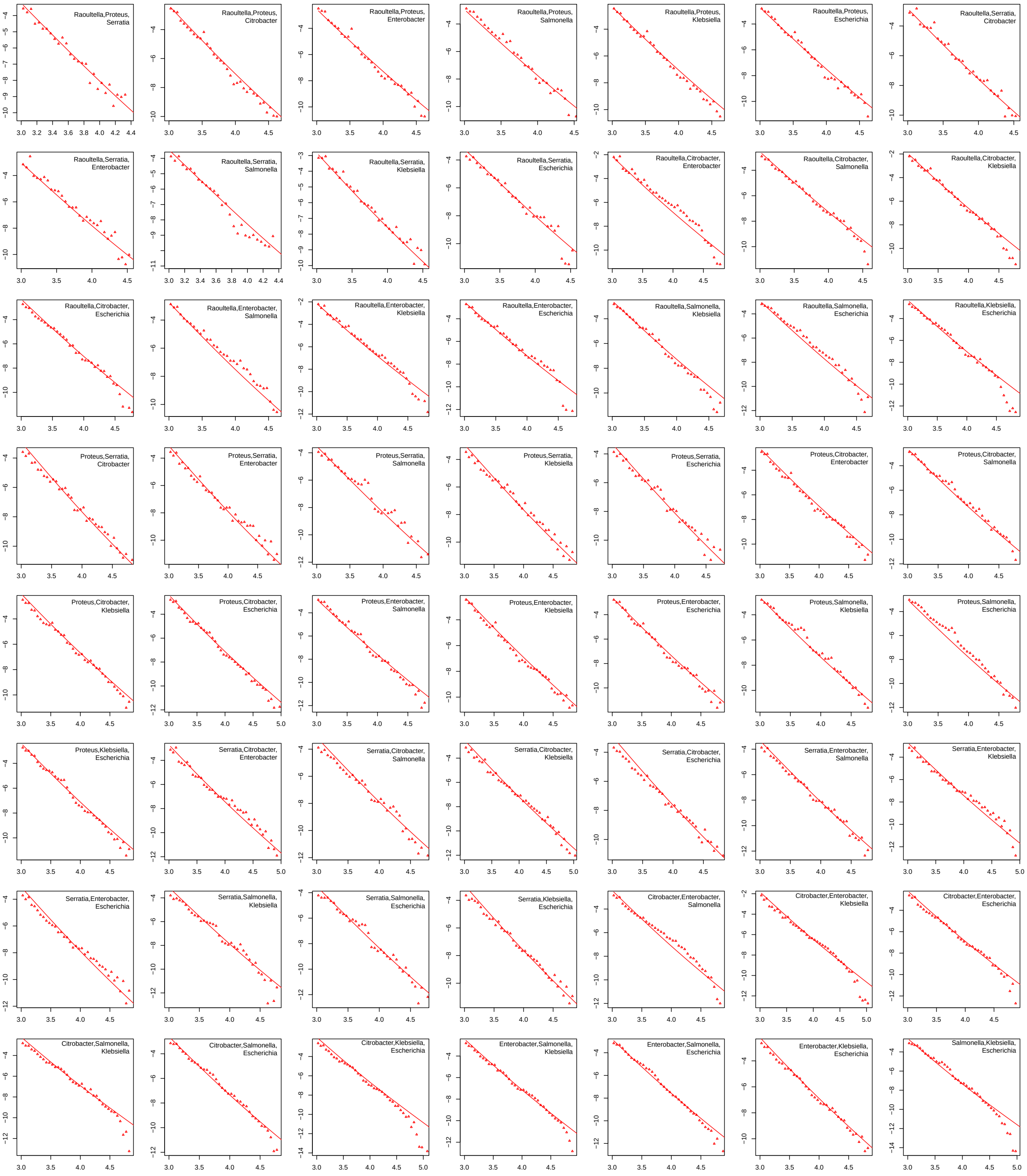

Figure S6: Here MLDs for $n = 3$ sets are presented on the $\log - \log$ scale: $\log_{10} m(r)$ vs. $\log_{10} r$. The sets are indicated in the upper right corners. Points represent the empirical data, while the lines represent the prediction of the model.

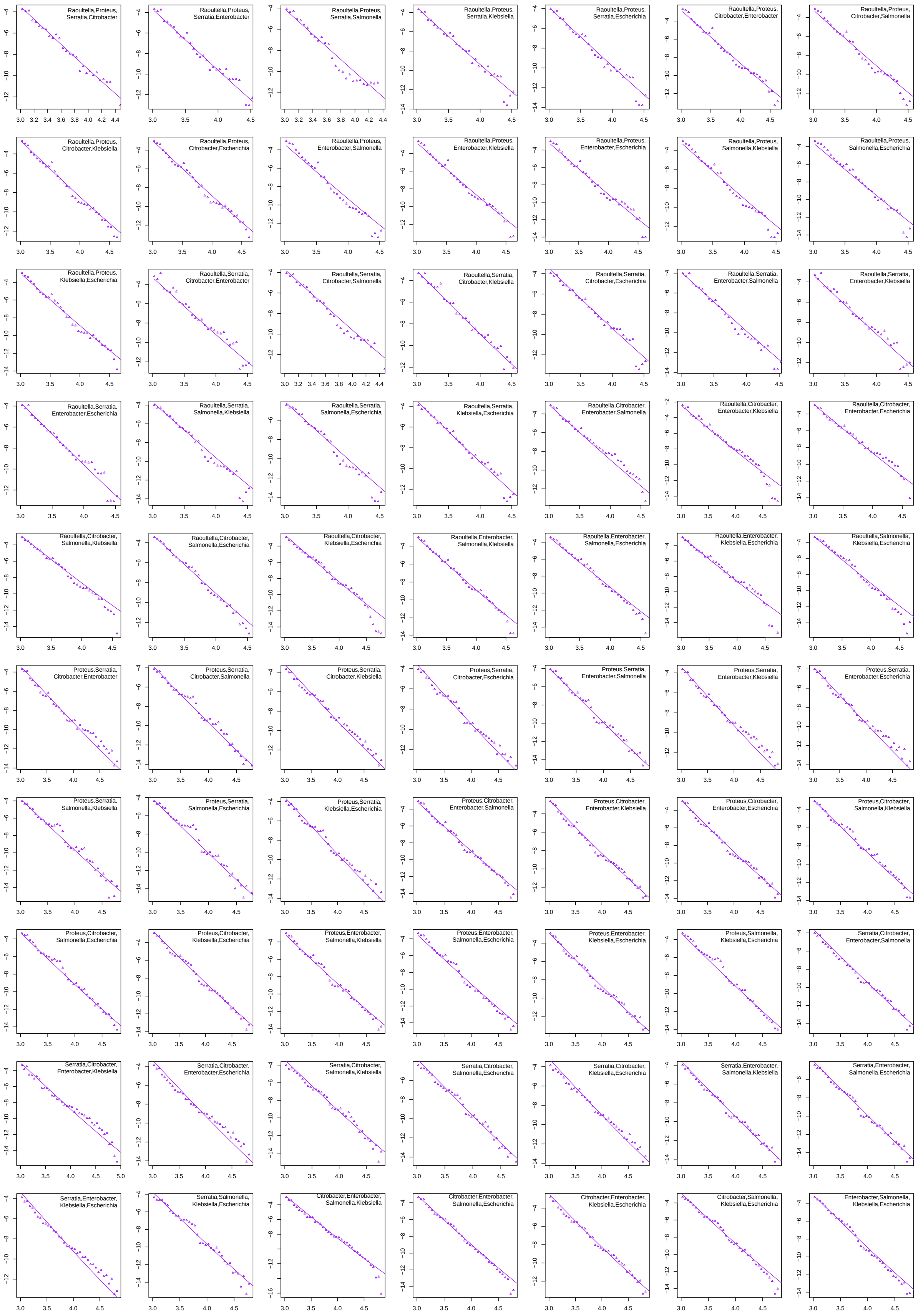

Figure S7: Here MLDs for $n = 4$ sets are presented on the $\log - \log$ scale: $\log_{10} m(r)$ vs. $\log_{10} r$. The sets are indicated in the upper right corners. Points represent the empirical data, while the lines represent the prediction of the model.

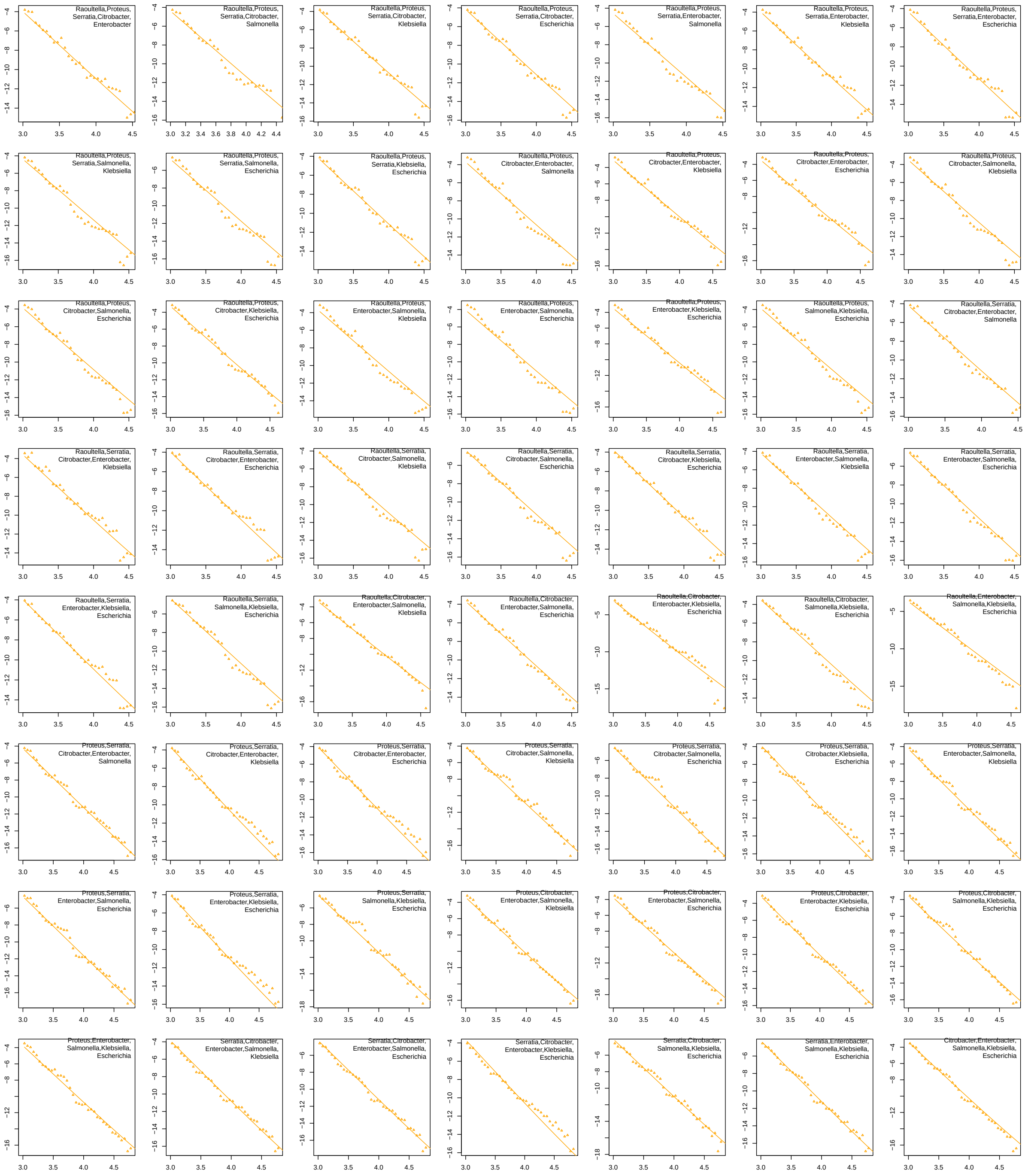

Figure S8: Here MLDs for $n = 5$ sets are presented on the log − log scale: $\log_{10} m(r)$ vs. $\log_{10} r$. The sets are indicated in the upper right corners. Points represent the empirical data, while the lines represent the prediction of the model.

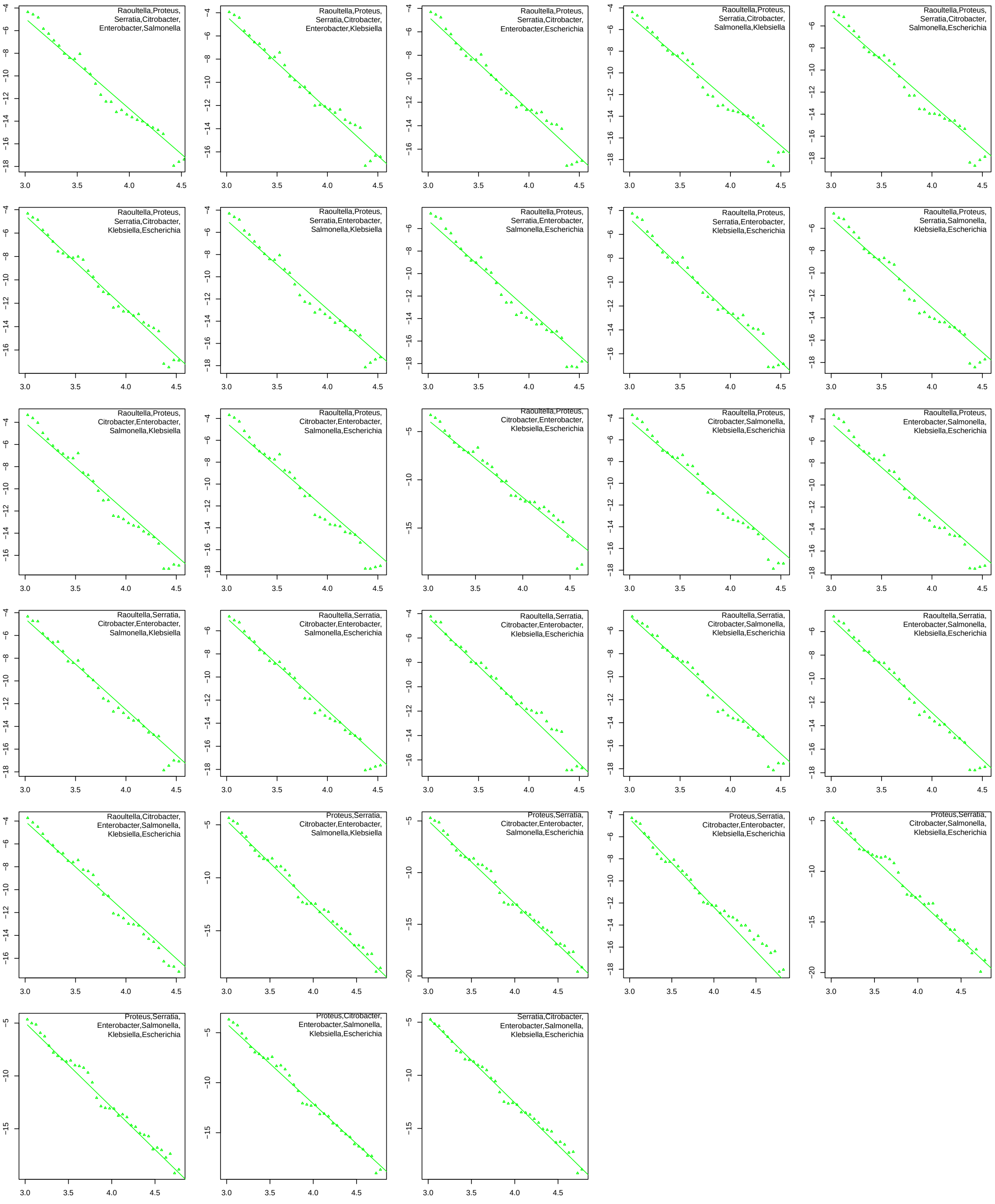

Figure S9: Here MLDs for $n = 6$ sets are presented on the log − log scale: $\log_{10} m(r)$ vs. $\log_{10} r$. The sets are indicated in the upper right corners. Points represent the empirical data, while the lines represent the prediction of the model.

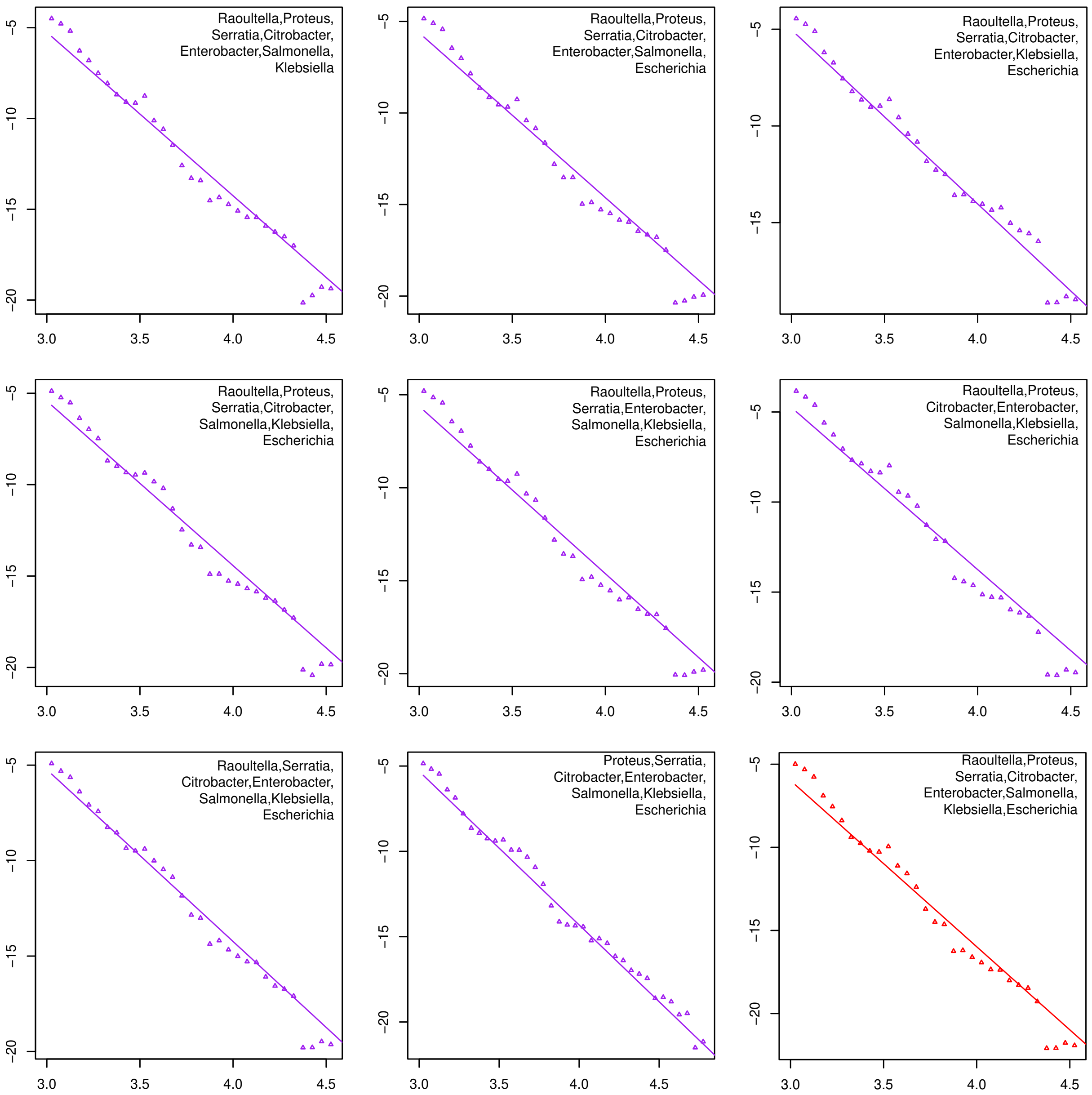

Figure S10: Here MLDs for $n = 7$ and $n = 8$ (last plot) sets are presented on the $\log - \log$ scale: $\log_{10} m(r)$ vs. $\log_{10} r$. The sets are indicated in the upper right corners. Points represent the empirical data, while the lines represent the prediction of the model.

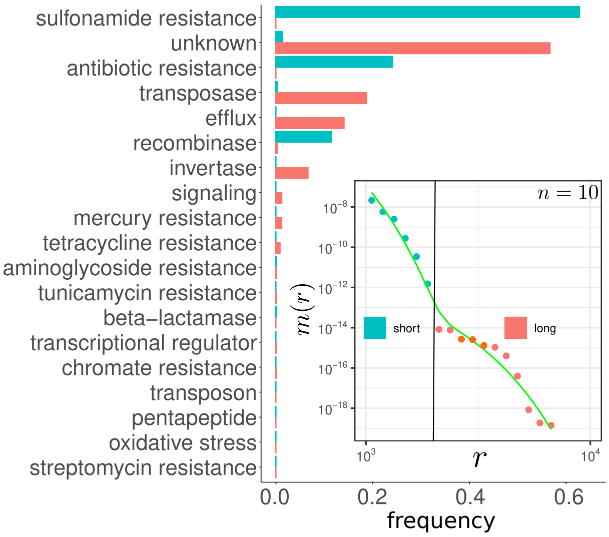

Figure S11: Functional analysis of matches shared by 10 genera: *Escherichia, Klebsiella, Salmonella, Enterobacter, Citrobacter, Serratia, Proteus, Raoultella, Vibrio* and *Cronobacter*. The MLD of the matches can be fitted with two exponential functions (see inset and Fig. 4(i)). Short matches (green dots and bars) and long matches (red dots and bars) have different functional annotations.

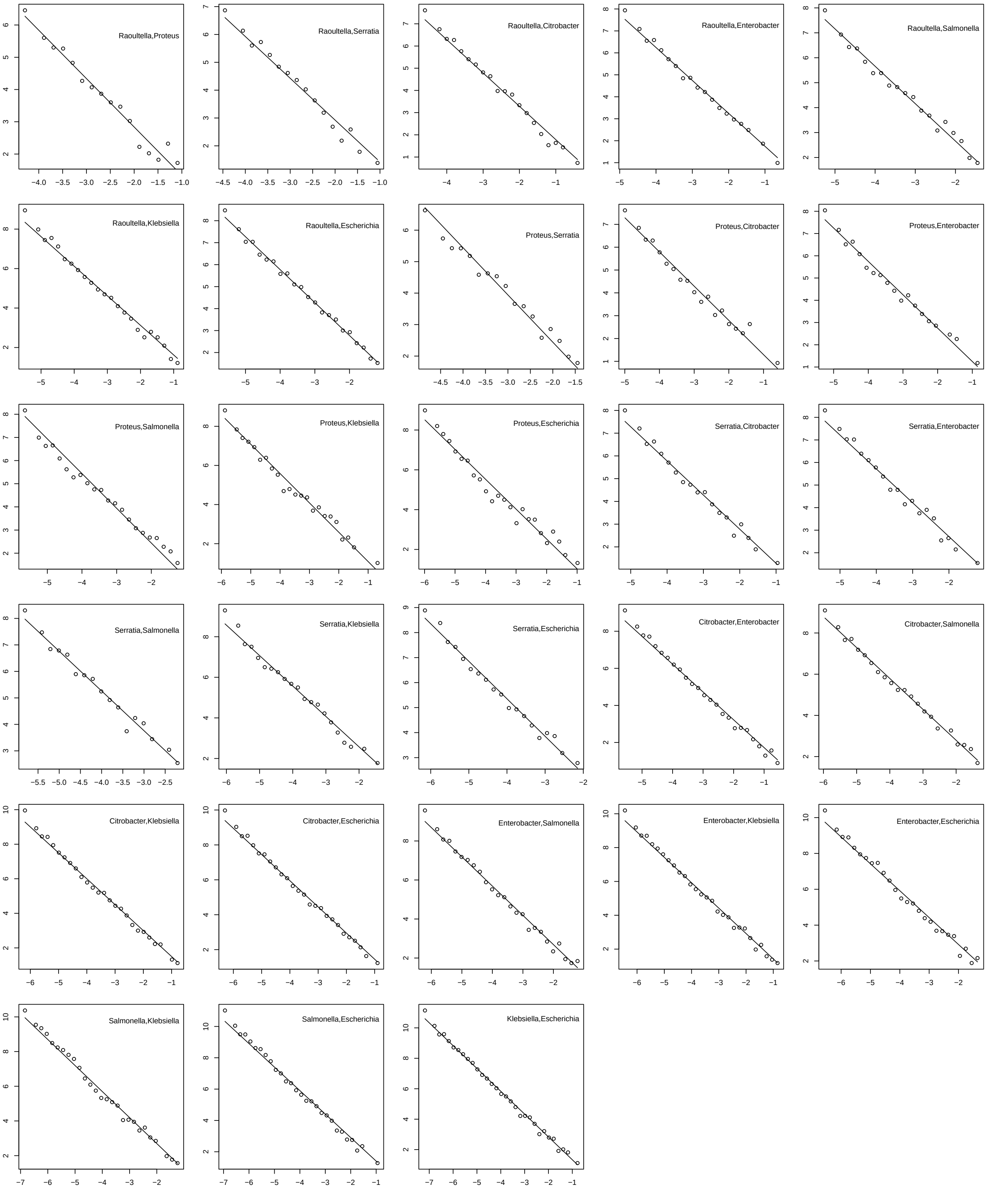

Figure S12: Here distributions of sequences abundances fractions are presented for $n = 2$. After clustering the matches we calculate the sizes of the clusters and divide by the total number of comparisons for each set (product of number of genomes for each genus in the set) to get the abundance fraction of each sequence—the probability to obtain this sequence taking $n = |s| = 2$ random genomes from the set s, each genome from a different genus. The probability density of this abundance fraction is presented on the plots for all pairs of genera (annotated in the upper right corners) on the $\log_{10} - \log_{10}$ scale. Points represent the empirical data, while the lines represent the power-law with $-3/2$ exponent (we don't have a theory that predicts this exponent).

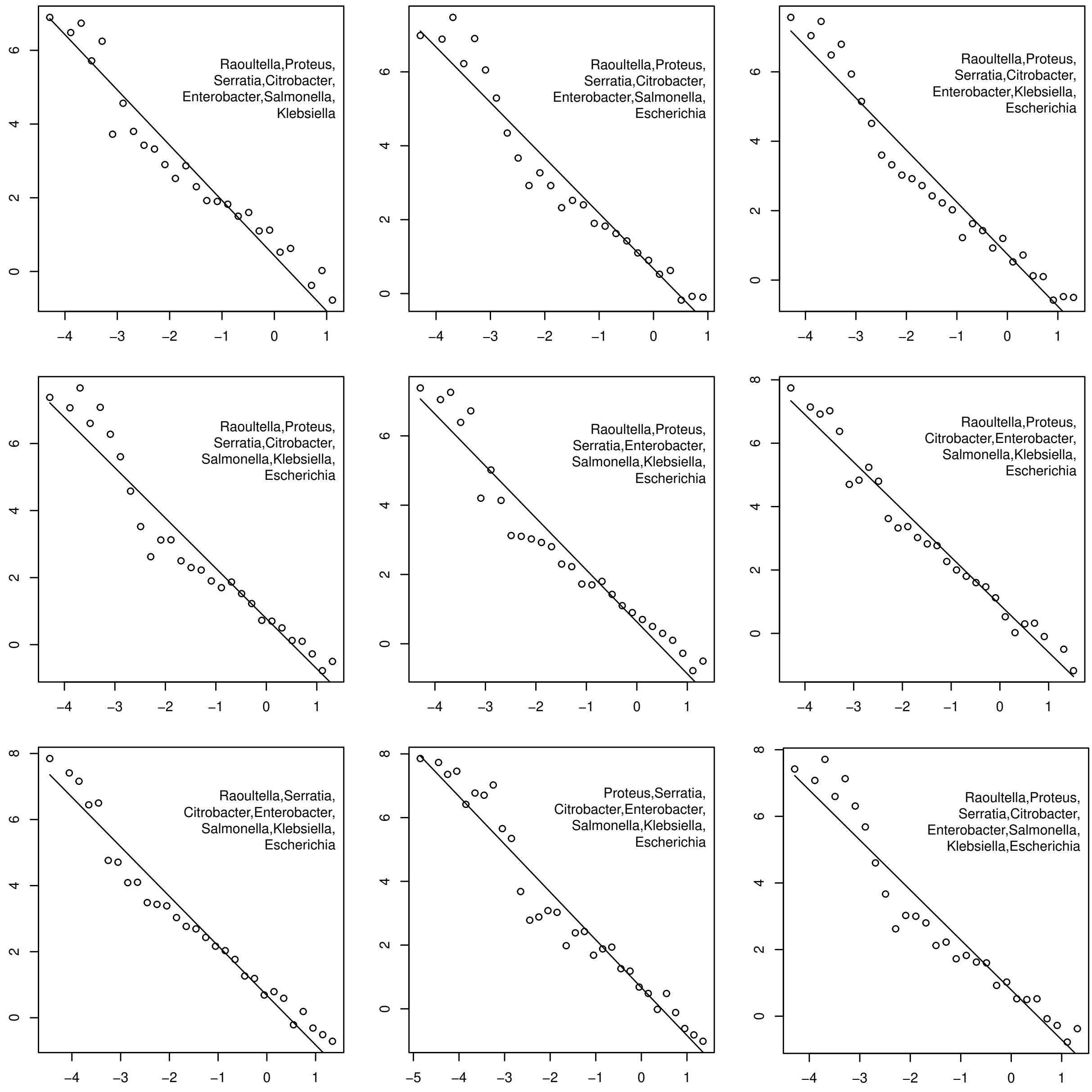

Figure S13: The same as in Fig. S12, but for sets of genera with $n = |s| = 7$ and 8 (last plot).

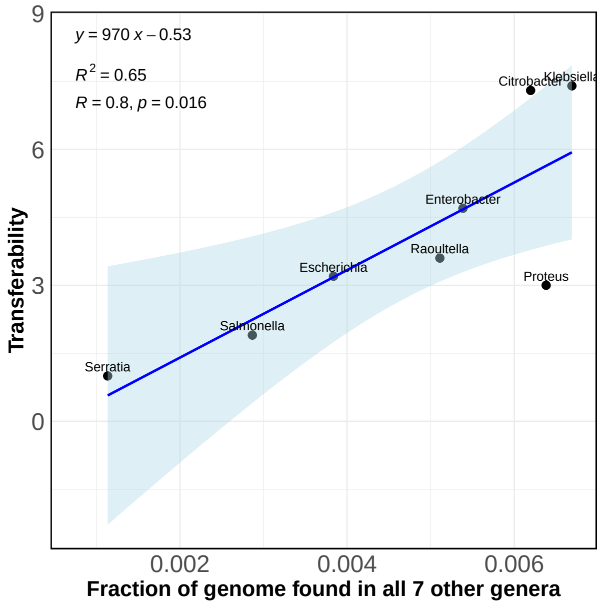

Figure S14: Calculated transferabilities of the eight studied genera *vs.* average fraction of genome shared with 7 other genera (at least one genome for each genus).

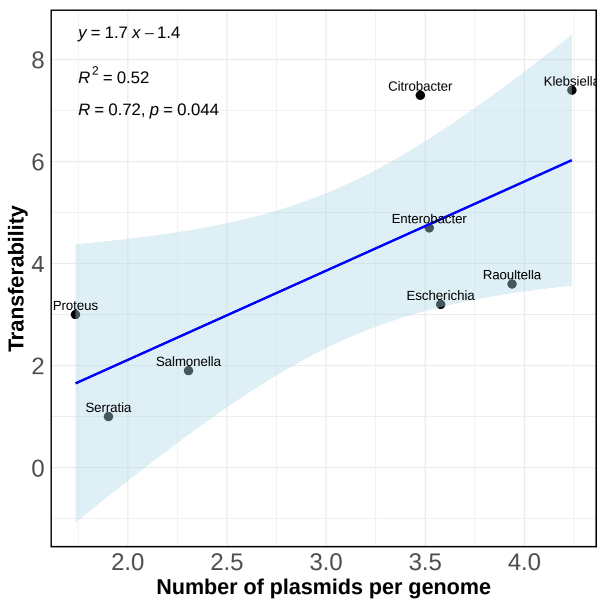

Figure S15: Calculated transferabilities of the eight studied genera *vs.* average number of plasmids per genome.
